# “Proton-Iodine” Regulation of Protonated Polyaniline Catalyst for High-Performance Electrolytic Zn-I_2_ Batteries

**DOI:** 10.1007/s40820-025-01928-5

**Published:** 2025-11-01

**Authors:** Mengyao Liu, Kovan Khasraw Abdalla, Meng Xu, Xueqian Li, Runze Wang, Qi Li, Xiaoru Zhang, Yanan Lv, Yueyang Wang, Xiaoming Sun, Yi Zhao

**Affiliations:** 1 State Key Laboratory of Chemical Resource Engineering, Beijing Advanced Innovation Center for Soft Matter Science and Engineering, College of Chemistry, Beijing University of Chemical Technology, Beijing, 100029 People’s Republic of China; 2 Beijing Key Laboratory of Environmental Science and Engineering, School of Materials Science and Engineering, Beijing Institute of Technology, Beijing, 100081 People’s Republic of China; 3 Shandong Key Laboratory of Advanced Chemical Energy Storage and Intelligent Safety, Advanced Technology Research Institute, Beijing Institute of Technology, Jinan, 250300 People’s Republic of China

**Keywords:** Electrolytic Zn-I_2_ battery, Proton-iodine regulation, Direct I^0^/I^−^ reaction conversion, Limited polyiodide shuttling, High performance

## Abstract

Low-cost and high-safety aqueous Zn-I_2_ batteries attract extensive attention for large-scale energy storage systems. However, polyiodide shuttling and sluggish iodine conversion reactions lead to inferior rate capability and severe capacity decay. Herein, a three-dimensional polyaniline is wrapped by carboxyl-carbon nanotubes (denoted as C-PANI) which is designed as a catalytic cathode to effectively boost iodine conversion with suppressed polyiodide shuttling, thereby improving Zn-I_2_ batteries. Specifically, carboxyl-carbon nanotubes serve as a proton reservoir for more protonated –NH^+^ = sites in PANI chains, achieving a direct I^0^/I^−^ reaction for suppressed polyiodide generation and Zn corrosion. Attributing to this “proton-iodine” regulation, catalytic protonated C-PANI strongly fixes electrolytic iodine species and stores proton ions simultaneously through reversible –N = /–NH^+^– reaction. Therefore, the electrolytic Zn-I_2_ battery with C-PANI cathode exhibits an impressive capacity of 420 mAh g^−1^ and ultra-long lifespan over 40,000 cycles. Additionally, a 60 mAh pouch cell was assembled with excellent cycling stability after 100 cycles, providing new insights into exploring effective organocatalysts for superb Zn-halogen batteries.

**Supplementary Information:**

The online version contains supplementary material available at 10.1007/s40820-025-01928-5.

## Highlights

- A three-dimensional polyaniline wrapped by carboxyl-carbon nanotubes (denoted as C-PANI) is designed as a catalytic cathode to effectively boost direct I^0^/I^−^ conversion for improved Zn-I_2_ batteries.
- Carboxyl-carbon nanotubes serve as a proton reservoir for more protonated –NH^+^ = sites in PANI chains, achieving “proton-iodine” regulation for suppressed polyiodide shuttling and Zn corrosion.
- Electrolytic Zn-I_2_ battery with C-PANI cathode exhibits an impressive capacity of 420 mAh g^−1^ and ultra-long lifespan over 40,000 cycles.

**Supplementary Information:**

The online version contains supplementary material available at 10.1007/s40820-025-01928-5.

## Graphical abstract

**Figure:**
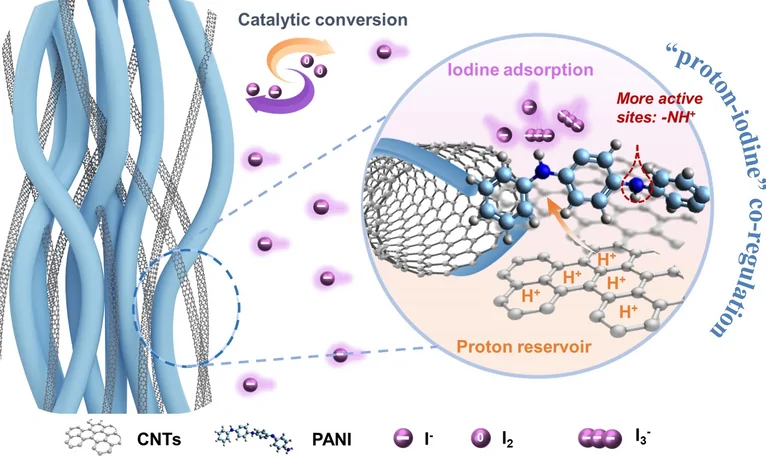

## Introduction

Aqueous Zn batteries are extensively explored in the pursuit of high-safe, low-cost, and large-scale energy storage systems, thus satisfying the increasing utilization demands of renewable energy [1, 2]. To date, manganese oxides, vanadium oxides, and Prussian blue analogs have been developed as cathode materials to enhance battery performance. Different from these conventional reported cathodes, iodine stands out as an emerging and highly promising cathode because of its high theoretical capacity (211 mAh g^−1^), flat discharge platform (1.38 V *vs.* Zn/Zn^2+^), and abundance in seawater (55 μg L^−1^) [3, 4]. However, iodine cathodes typically suffer from challenges of poor conductivity, polyiodide shuttling, and sluggish iodine conversion kinetics, leading to inferior rate capability and serious capacity decay [5]. More seriously, the shuttled polyiodide ions can react with the Zn anode like “Zn + I_3_^−^  → Zn^2+^  + 3I^−^,” thus causing uncontrolled self-discharge and Zn corrosion issues [6]. The challenges faced by Zn-I_2_ batteries are intricate and correlative, thus resolving them through a facile but effective strategy is urgent toward the fast-charging and durable Zn-I_2_ batteries.

Ongoing research and endeavors are intently centered on optimizing iodine-host materials, including porous carbon matrix, starch, layered double hydroxides, and metal-nitrogen-doped carbon (M–N–C), restricting the polyiodide shuttle effect and improving cathodic conductivity [7–10]. Yet, the finite physical and/or chemical adsorption with iodine species is inadequate for high-efficient iodine conversion, especially in high-load iodine cathodes, thereby leading to low iodine utilization and modest capacity [11, 12]. Notably, toxic iodine vapors or volatile iodine solutions with high concentration have to be widely used to fabricate the above-mentioned I_2_-based cathodic composites, bringing safety and environmental pollution issues [13, 14]. To better intercept the polyiodide migration and shield Zn anodes from reactive iodine species, constructing protective layers onto Zn surface (e.g., metal–organic frameworks), introducing electrolytic additions (e.g., vermiculites nanolayers, hydrogels), as well as modifying separators (e.g., Janus separator) have been reported as effective approaches [15–18]. However, these anodic protection and functional separators do not radically solve the polyiodide dissolution and shuttling problems. Moreover, electrolyte additives were typically developed to suppress polyiodide shuttling rather than inhibit their generation [19–21]. Additionally, highly concentrated additives may introduce concerns related to cost and safety. Recently, cation adsorption engineering of trimethylsulfonium cation (TMS^+^) and Fe-N_4_ sites in single-atom Fe-NC wrapped high-area carbon (HC@FeNC) was explored to effectively fix electrolytically active I^−^ and trap polyiodide onto the cathodic carbon cloth to stabilize Zn-I_2_ batteries [22, 23]. Nevertheless, these non-redox-active TMS^+^ or HC@FeNC fails to further improve the energy density of Zn-I_2_ batteries. Based on this, conjugated polymers with abundant charged groups and lightweight have been studied as promising organic catalysts to enhance the adsorption ability and boost the conversion efficiency of iodine simultaneously.

Very recently, phenylamine-based conjugated microporous polymer (PA-CMP) and two-dimensional Zn-meso-5,10,15,20-tetra-kis (4-carboxyphenyl) porphyrin (Zn-TCPP) with secondary amines and porphyrin nitrogen, respectively, were developed to improve I^−^/I_3_^−^/I_5_^−^ conversion kinetics in ZnI_2_ or KI-containing electrolytes [24, 25]. Moreover, poly(1,5-naphthalenediamine, NDA) was introduced into the VO_2_ cathode to enable the hierarchical NDA@VO_2_ cathode with additional ~ 200 mAh g^−1^ induced by the co-reactions of –C = N/–NH_2_ and I^−^/I^0^ [26]. Yet, the synthetic process of these catalytic organics is complicated to realize the target of high-capacity Zn-I_2_ batteries. Based on this issue, typical polyaniline (PANI) was utilized as the catalytic cathode to boost I^−^/I_3_^−^ conversion efficiency along with the redox chemistry of –N = /–NH^+^- in 1 M ZnI_2_ + 9 M ZnCl_2_ or 1 M ZnAc_2_ + 4 M NH_4_I electrolytes [27, 28]. However, the highly concentrated halogen ions are a disadvantage to the ionic conductivity, Zn anode stability, and battery cost-effectiveness. More seriously, bare PANI hardly releases their redox potential and supplies protonated -NH^+^- sites caused by the low proton concentration in mild-acid electrolytes [29]. These protonated -NH^+^- groups are effective for the adsorption and capture of iodine species, thus improving the iodine utilization ratio and redox kinetics. Designing the protonation of polyaniline was deliberately employed to accelerate PANI redox kinetics and increase the density of active sites, thereby synergistically modulating iodine catalysis and enhancing the overall energy storage capacity of the batteries [30, 31]. Therefore, proton-reservoir strategies should investigate the “proton-rich” PANI catalyst to boost iodine conversion and trap polyiodide for high-rate and stable Zn-I_2_ batteries.

**Scheme 1:**
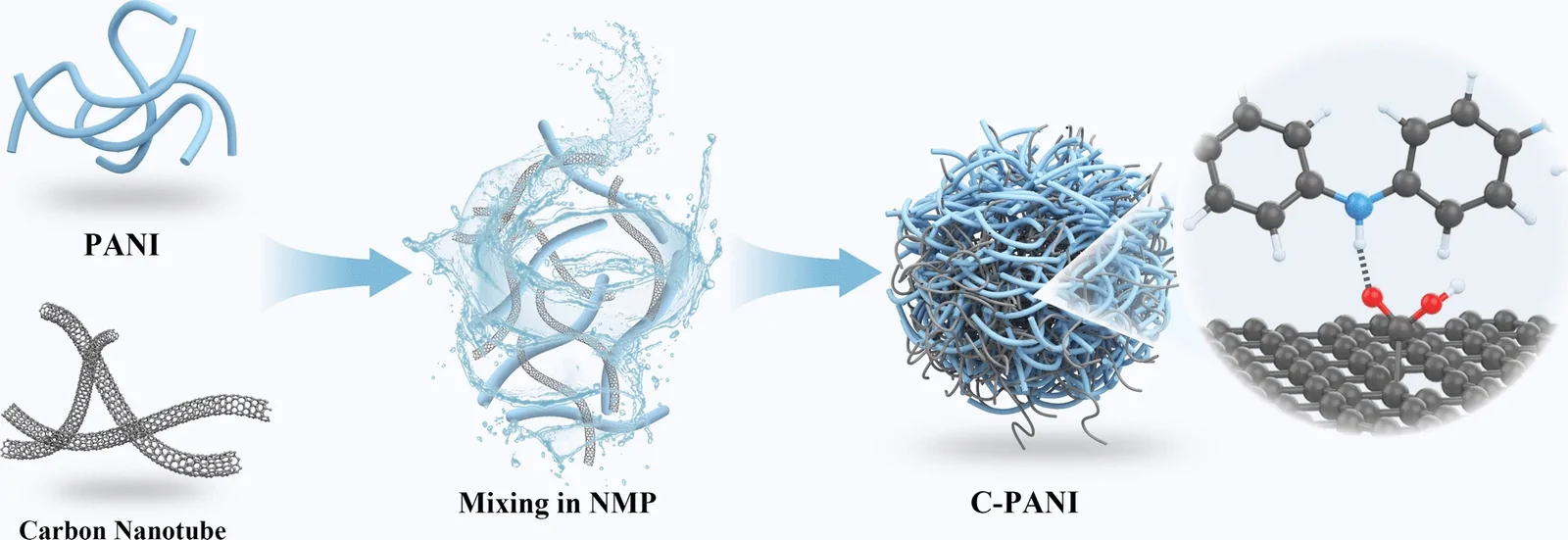
Schematic synthesis diagram of catalytic C-PANI as a high-efficient and robust cathode for electrolytic Zn-I_2_ batteries

Inspired by this, we presented a synergistic “proton-iodine” strategy to improve the iodine adsorption and catalytic conversion capabilities of carboxyl-carbon nanotubes wrapped PANI (denoted as C-PANI) as the catalytic cathode for high-performance Zn-I_2_ batteries within 2 M Zn(OTF)_2_/0.3 M NH_4_I electrolyte. Specifically, PANI nanorods were incorporated into carboxyl-carbon nanotubes (carboxyl-CNTs) uniformly through a facile solvothermal stirring method (Scheme 1). Owing to the negative-charged carboxyl groups, carboxyl-CNTs can effectively fix the proton ions from the electrolyte and construct “proton-rich” localization within C-PANI electrodes to greatly stimulate reactivity and stability of C-PANI in a mild-acid electrolyte. More importantly, protonated PANI featuring abundant –NH^+^ = and –NH^+^- sites can function as a polyiodide binder to realize direct I^0^/I^−^ conversion and suppress polyiodide shuttling simultaneously, thereby boosting reaction kinetics and inhibiting Zn corrosion. Benefiting from the “proton-iodine” co-regulation of C-PANI electrodes, the resulting Zn-I_2_ battery delivers an ultra-high capacity of 423 mAh g^−1^ at 1 A g^−1^ and excellent cycling stability after 40,000 cycles at 20 A g^−1^. Furthermore, the high-load pouch cell demonstrates 90.8% capacity retention, maintaining 60 mAh after 100 cycles. This study paves novel insights into exploring highly-efficient organocatalysts for advanced Zn-I_2_ batteries with a dual-energy storage mechanism.

## Results and Discussion

### Design Principle and Structural Characterizations

**Fig. 1:**
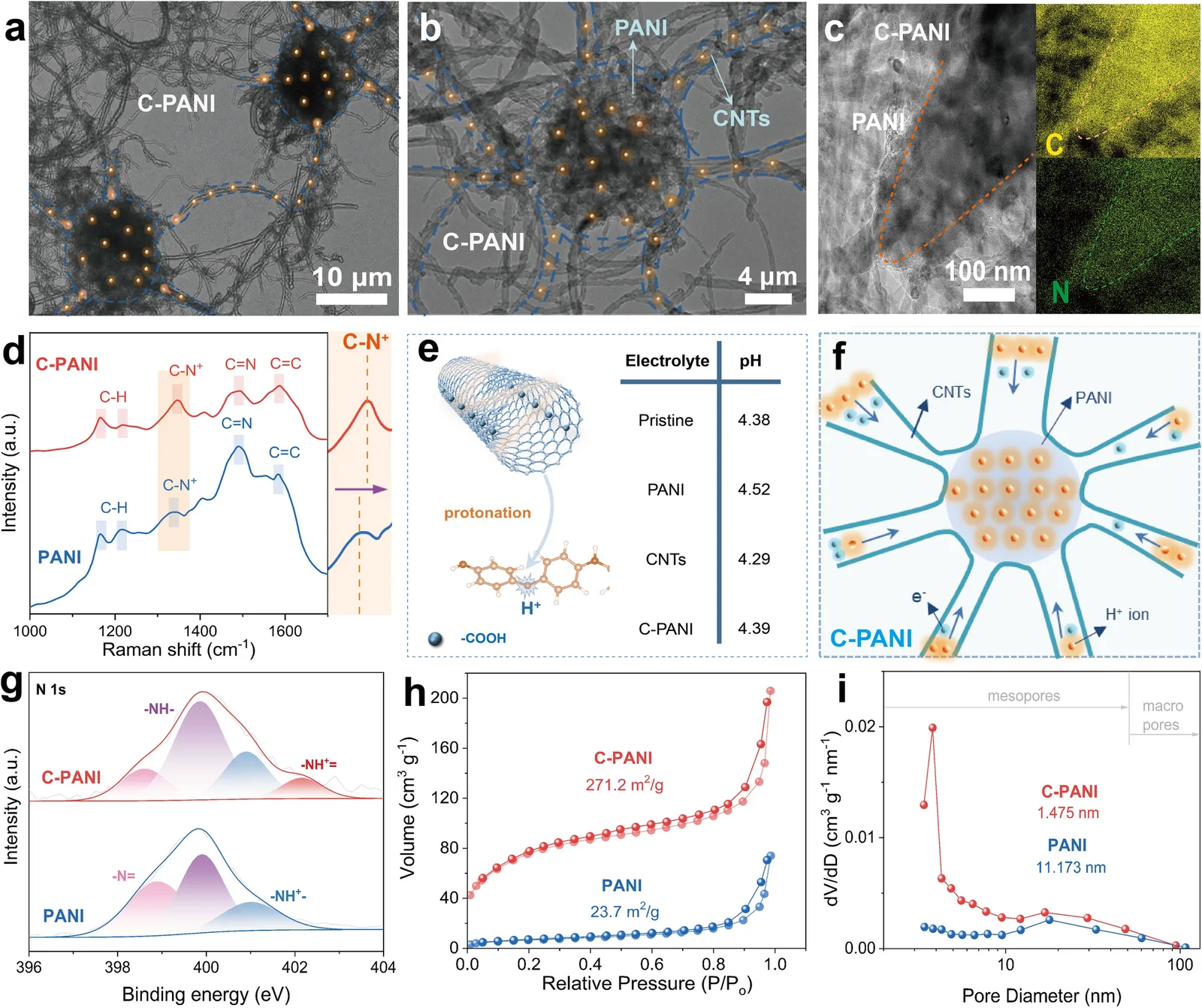
Structural characterizations of C-PANI, PANI, and carboxyl-CNTs. **a**, **b** TEM images of C-PANI powder. **c** Elemental mapping analysis of C-PANI powder. **d** Raman spectra of the PANI and C-PANI. **e** pH value changes after adding three different cathodes to 2 M Zn(OTF)_2_/0.3 M NH_4_I electrolyte. **f** A schematic diagram of the neuron-inspired C-PANI with the protonated PANI contributes to carboxyl-CNTs. **g** XPS N 1*s* spectra, **h** N_2_ adsorption/desorption isotherm curves, and **i** Pore size distribution curves of PANI and C-PANI, respectively

In this work, C-PANI powder was obtained by a scalable solvothermal stirring-grinding method to realize the uniform mixture of PANI and carboxyl-CNTs with a “proton-rich” environment. Figure S1 displays the obtained 30 g C-PANI mixture for the large-size catalytic cathode of Zn-I_2_ batteries. The transmission electron microscopy (TEM) images of C-PANI successfully suggested the well-formed conductive networks of PANI nanorods tightly trapped by carboxyl-CNTs (Fig. 1a, b). The neural network-like architecture of C-PANI establishes a percolated conductive pathway throughout the electrode, dramatically lowering electron transfer and charge diffusion resistance for boosted rate capability. The energy-dispersive X-ray spectroscopy (EDS) images further demonstrated that PANI with N atoms was covered by the carboxyl-CNTs (Fig. 1c). Such an organic–inorganic structure enables the C-PANI host with a more reactive electrode/electrolyte interface, boosting charge transfer kinetics. More morphology and structural details of PANI and C-PANI with different magnifications can be seen in Figs. S2 and S3. Besides, the X-ray diffraction (XRD) patterns of PANI and C-PANI indicated the successful combination of PANI and carboxyl-CNTs (Fig. S4) [32]. Compared with bare PANI, the Raman spectrum of C-PANI showed a redshift of the C-N^+^ peak caused by the hydrogen bonds and π–π interactions between PANI and carboxyl-CNTs, indicating the boosted -C = N (1467 cm^−1^) → C-N^+^ reaction for protonated PANI chains (Fig. 1d) [33, 34]. Moreover, the comparison of pH changes after the addition of PANI, carboxyl-CNTs, and C-PANI, respectively, into the 2 M Zn(OTF)_2_/0.3 M NH_4_I electrolyte was conducted (Fig. 1e). Due to the proton confinement effect of carboxyl-CNTs, C-PANI displayed a practically negligible pH variation caused by the protonated PANI with enhanced redox activity. Specifically, the neuron-inspired C-PANI cathode with well-formed carboxyl-CNTs networks exhibits boosted charge and electron transfer kinetics, particularly proton and iodine ions, which is essential for improved Zn-I_2_ batteries (Fig. 1f). Compared to bare PANI, C-PANI displayed decreased -N = (398.6 eV) as well as increased -NH^+^ = (402.3 eV) and -NH^+^- (401 eV) peaks, reconfirming the protonation of C-PANI with improved reactivity for strong iodine anchoring and conversion effect (Fig. 1g) [29]. According to the N_2_ adsorption/desorption isotherm analyses, C-PANI presents a higher specific surface value of 271.2 m^2^ g^−1^ than that of PANI (23.7 m^2^ g^−1^), endowing C-PANI with better electrode/electrolyte interaction and iodine storage ability (Fig. 1h). Moreover, the average pore size of C-PANI (1.475 nm) was much smaller compared with bare PANI (11.173 nm), which further suggested its inhibited polyiodide shuttling for high-efficient iodine conversion reaction (Fig. 1i). Therefore, neural network-like C-PANI with abundant -NH^+^-/-NH^+^ = groups exhibits “proton-iodine” regulation for enhanced iodine catalytic conversion ability.

### Electrochemical Performance of Zn-I_2_ Batteries

**Fig. 2:**
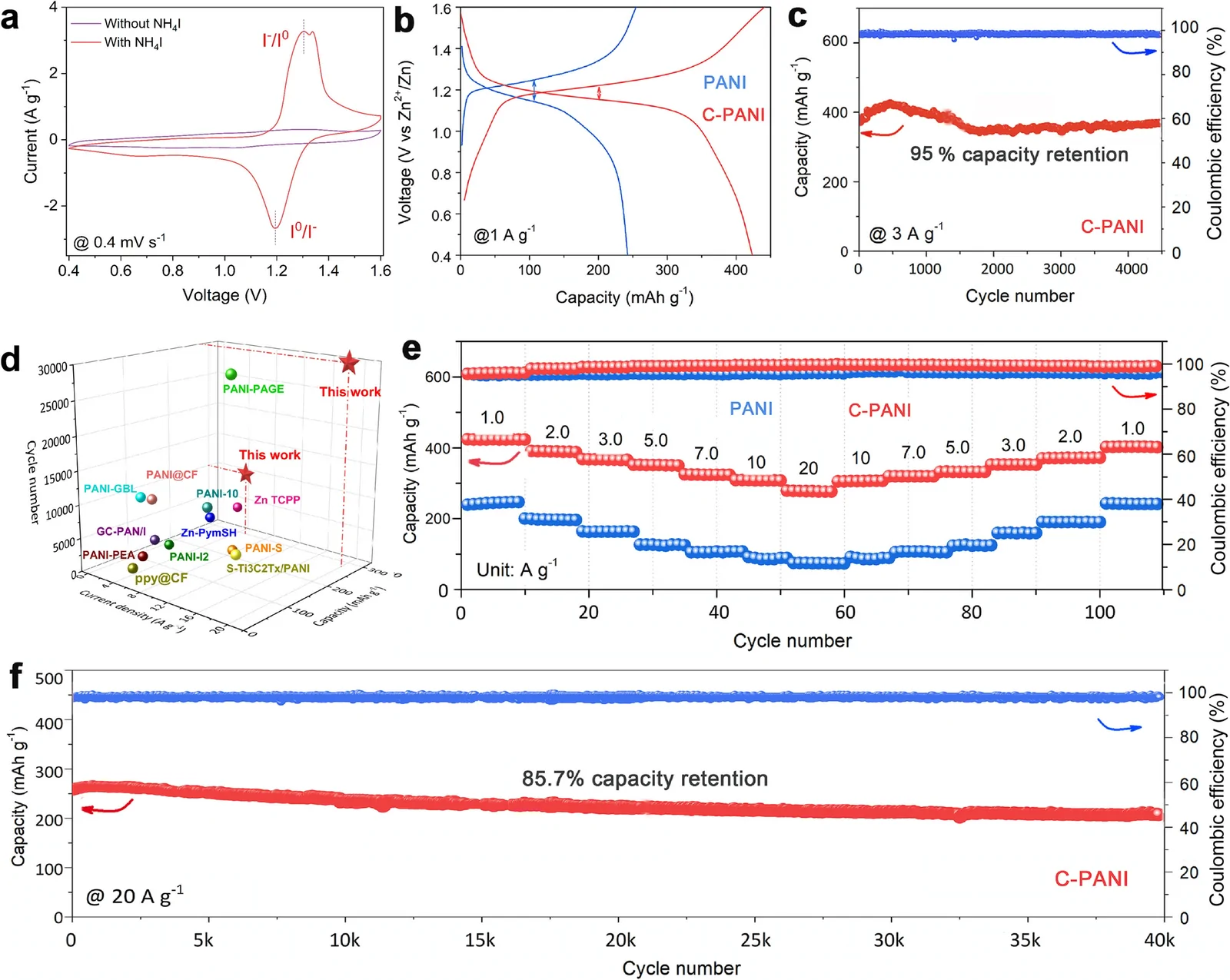
Electrochemical performance of assembled Zn-I_2_ batteries based on C-PANI and PANI cathodes in 2 M Zn(OTF)_2_/0.3 M NH_4_I electrolyte. **a** The second cycle CV curves of C-PANI in 2 M Zn(OTF)_2_ with or without 0.3 M NH_4_I electrolytes. **b** GCD profiles of C-PANI at 1 A g^−1^. **c** Cycling performances of C-PANI at 3 A g^−1^. **d** Battery performance comparison of C-PANI and other electrolytic cathodes. **e** Rate performances of C-PANI at different current densities. **f** Cycling performances of C-PANI at 20 A g^−1^

In conventional Zn-I_2_ batteries, porous carbon/I_2_ cathode materials typically face inferior rate capability and poor stability caused by the sluggish iodine conversion kinetics and severe polyiodide shuttling [7, 10]. To address this, redox-active C-PANI hosts with the strong physical-chemistry adsorption ability are applied as the redox-active organocatalyst for electrolytic Zn-I_2_ batteries with 2 M Zn(OTF)_2_/0.3 M NH_4_I electrolyte. To obtain optimal battery performance, we systematically evaluated a range of testing conditions, including adjusting the carbon matrix types, electrode mass ratio, and NH_4_I concentration (Figs. S5-S7). As a result, the mass ratio of carboxyl-CNTs/PANI/super P was chosen as 6:3:1 for C-PANI cathode within 0.3 M NH_4_I addition for the following battery tests. In this setup, C-PANI maintained 155 mAh g^−1^ after 1000 cycles at 0.2 A g^−1^ in 2 M Zn(OTF)_2_ electrolyte, indicating the robust but efficient redox-catalytic ability of C-PANI for high-performance Zn-I_2_ batteries (Fig. S8). Compared with bare PANI, the C-PANI cathode showed much higher capacity retention of 325 mAh g^−1^ after 600 cycles at 1 A g^−1^, suggesting its robust catalytic ability for iodine conversion reaction (Fig. S9). To better investigate the catalytic activity, cyclic voltammetry (CV) tests of the C-PANI electrode were carried out with or without 0.3 M NH_4_I addition, respectively. In 2 M Zn(OTF)_2_/0.3 M NH_4_I electrolyte, newly formed redox peaks were observed for the C-PANI cathode at around 1.30/1.20 V, indicating the reversible oxidation and reduction of I^−^/I^0^ during battery working (Figs. 2a and S10) [23]. Moreover, galvanostatic charge/discharge (GCD) profiles demonstrated that C-PANI cathode delivered higher capacity of 420 mAh g^−1^ (0.80 mAh cm^−2^) and lower polarization voltage than that of bare PANI (245 mAh g^−1^, 0.47 mAh cm^−2^) at 1 A g^−1^, illustrating the boosted iodine conversion efficiency and reaction kinetics catalyzed by C-PANI (Fig. 2b). The enhanced battery performance of C-PANI can be further manifested in its rate capability and cycling stability. As depicted in Fig. 2c, the C-PANI cathode displayed an initial discharge capacity of 368 mAh g^−1^ and an outstanding capacity retention rate of 98% after 4500 cycles at 3 A g^−1^. Moreover, C-PANI electrode showed superior rate capacities of 423, 390, 363, 350, 323, 308, and 277 mAh g^−1^ at 1, 2, 3, 5, 7, 10, and 20 A g^−1^, respectively, than that of PANI (244, 196, 163, 124, 105, 85, and 74 mAh g^−1^), as shown in Fig. 2e. More details of the GCD profiles at different current densities are displayed in Fig. S11. Such outstanding rate performance of C-PANI can be attributed to the more accessible active sites (-NH^+^ = and -NH^+^-) and charge transfer pathways introduced by the connected carboxyl-CNTs.

In addition, the long-term cycling performance of the C-PANI cathode was more remarkable than that of bare PANI, confirming the strong iodine fixation capability and inhibited polyiodide shuttling of C-PANI. Specifically, the C-PANI electrode maintained high discharge capacity of 320 mAh g^−1^ (5 A g^−1^) and 280 mAh g^−1^ (10 A g^−1^) after 10,000 cycles and 20,000 cycles, respectively (Figs. S12 and S13). However, bare PANI cathodes underwent significant capacity deterioration and operated approximately 6,700 and 10,500 cycles before battery failure. Additionally, the C-PANI electrode displayed an initial capacity of 260 mAh g^−1^ and maintained a high-capacity retention rate of 85.7% after 40,000 cycles at 20 A g^−1^, suggesting the preeminent catalytic activity of redox-catalytic C-PANI cathode (Fig. 2f). Notably, bare PANI and carboxyl-CNTs electrodes displayed low capacity and inferior cycling stability in 2 M Zn(OTF)_2_/0.3 M NH_4_I electrolyte at 20 A g^−1^, reconfirming the synergistic effect of PANI and carboxyl-CNTs in iodine conversion redox and polyiodide shuttle inhibition (Fig. S14). Compared with reported organic and organic/iodine-based cathodes, the exceptional capacity retention and lifespan of the catalytic C-PANI cathode proved the firm interaction between the iodine species and -NH^+^-/-NH^+^ = sites for fast-charging and robust Zn-I_2_ batteries (Fig. 2d and Table S1) [25, 27–30, 35–41].

### Iodine Conversion Kinetic Analysis of Zn-I_2_ Batteries

**Fig. 3:**
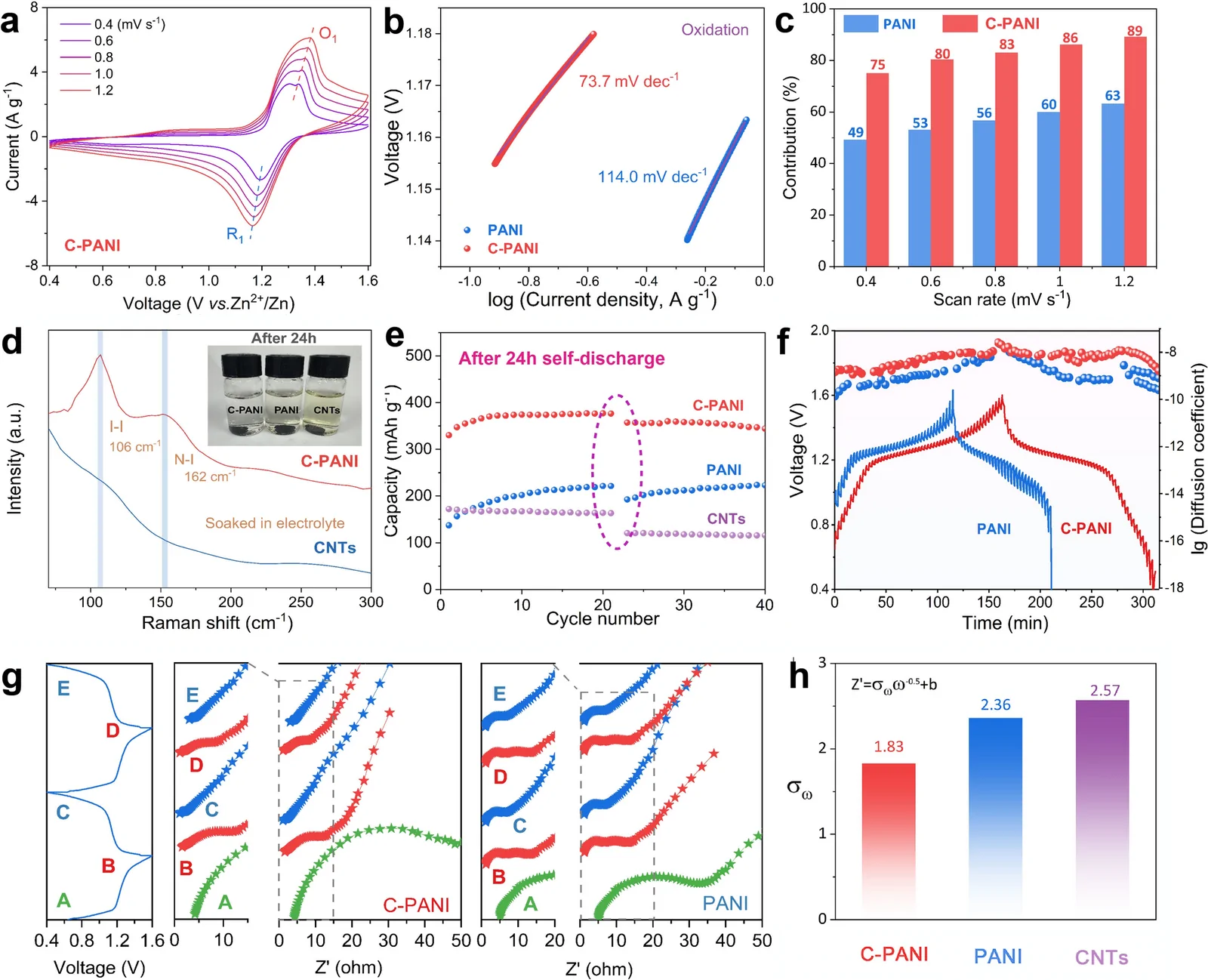
Kinetic analysis of redox reaction in assembled Zn-I_2_ batteries based on C-PANI, PANI, and CNTs cathodes, respectively, in 2 M Zn(OTF)_2_/0.3 M NH_4_I electrolyte. **a** CV curve of C-PANI at various scan rates. **b** Tafel plots of C-PANI and PANI cathodes during the iodine oxidation process, respectively. **c** Normalized contribution ratios of capacitive-controlled capacity at different scan rates. **d** Raman spectra of the three cathodes after 24-h immersion into 2 M Zn(OTF)_2_/0.3 M NH_4_I electrolyte. **e** Cycling performance after 24-h self-discharge test at 3 A g^−1^. **f** Discharge/charge GITT profiles and corresponding ionic diffusion coefficient. **g** In situ EIS plots during the initial two cycles of C-PANI and PANI cathodes. **h** Calculated Warburg impedance (σ_*w*_) at 0.4 V in the first cycle

To systematically evaluate the electrocatalytic behavior of the C-PANI electrode for the iodine conversion kinetics, a series of CV curves of the electrolytic Zn-I_2_ batteries were collected in 2 M Zn(OTF)_2_/0.3 M NH_4_I electrolyte (Fig. 3a). Comparing with bare carboxyl-CNTs and PANI, the catalytic C-PANI cathode displayed the lowest polarization potential between the anodic and cathodic peaks, suggesting its efficient oxidation/reduction reaction for iodine conversion (Fig. S15). The calculated *b* values of the redox peaks can be determined by the linear relationship of log(*i*) and log(*v*), where *i* and *v* are the peak current and scan rate, respectively. If *b* = 1, it indicates a capacitive-controlled process. While *b* = 0.5, it means a diffusion-controlled process [25]. As a result, C-PANI displayed the higher *b* values of oxidation (0.74) and reduction (0.81) peaks than that of the bare PANI electrode (0.57, 0.72), indicating a capacitive-controlled behavior for the C-PANI cathode (Fig. S16). In order to calculate the different current contributions, the current (*i*) is separated by the equation: *i* = k_1_*v* + k_2_*v*^1/2^, where k_1_*v* and k_2_*v*^1/2^ represent the current contributions from the capacitive effect and diffusion-controlled process, respectively [29]. Thus, the improved capacitive contribution ratios of C-PANI are 66%, 71%, 74%, 76%, and 79% at 0.4, 0.6, 0.8, 1.0, and 1.2 mV s^−1^, respectively, which are higher than that of bare PANI electrode and exceed that of the carboxyl-CNTs cathode (Figs. 3c and S17, S18). Such high-proportion capacitive contributions in the C-PANI cathode enable the high-rate performance of electrolytic Zn-I_2_ batteries. The Tafel slope (η) calculated from the CV profiles can reflect the reaction activity of a given cathode [35]. Remarkably, the C-PANI catalyst shows the much smaller η of 73.7 and 64.8 mV dec^−1^ for the iodine redox peaks compared with that of bare PANI (114.0 and 121.1 mV dec^−1^), confirming the boosted iodine conversion kinetics induced by “proton-iodine” regulation of C-PANI (Figs. 3b and S19).

The galvanostatic intermittent titration technique (GITT) was performed to further clarify the improved iodine redox kinetics of C-PANI through the calculated ionic value of diffusion coefficient (*D*) [29]. Comparing with that of PANI, C-PANI displayed an obviously higher *D* value between 10^−10^ and 10^−7^ cm^2^ s^−1^, suggesting the enhanced charge diffusion kinetics within the catalytic C-PANI cathode for efficient iodine redox (Fig. 3f). Besides, C-PANI electrode effectively minimized the capacity decay after the 24-h self-discharge process, indicating the strong iodine fixation ability and inhibited polyiodide shuttling caused by the protonated PANI chains in C-PANI cathode (Figs. 3e and S20). To better prove the iodine adsorption capability, bare PANI, carboxyl-CNTs, and C-PANI electrodes were immersed in 0.3 M NH_4_I solution, respectively (Fig. S21). Compared with the other two electrodes, C-PANI enabled the pristine yellow solution to become transparent after 24 h, indicating its strongest fixation ability for polyiodide ions. After iodine adsorption, the C-PANI electrode displayed distinct characteristic peaks of I-I (106 cm^−1^) and N-I (162 cm^−1^), whereas bare carboxyl-CNTs displayed none of these two peaks (Fig. 3d) [25]. Such a highly effective iodine capture property can be attributed to the characteristic C-N^+^ sites in protonated PANI chains within C-PANI [31]. In situ electrochemical impedance spectroscopy (EIS) spectra were further performed in the initial two cycles to highlight the catalytic iodine conversion effect of C-PANI (Figs. 3g and S22). Specifically, the fitted charge transfer resistance (R_ct_) and Warburg impedance (σ_*w*_) of C-PANI were smaller than that of PANI during battery cycling, indicating the “proton-iodine” effect induced by protonated PANI for boosted I^−^ adsorption/transportation (Figs. S23 and S24) [42]. Notably, C-PANI displayed non-obvious σ_*w*_ variation during cycling, suggesting its robust catalytic ability for reversible I^−^/I^0^ reaction. Besides, the σ_*w*_ of C-PANI (1.83) is smaller than that of PANI (2.36) and carboxyl-CNTs (2.57) at 0.4 V, confirming fast proton/iodine diffusion kinetics of C-PANI for high-rate Zn-I_2_ batteries (Fig. 3h).

### Reaction Mechanism of Assembled Zn–I_2_ Batteries

**Fig. 4:**
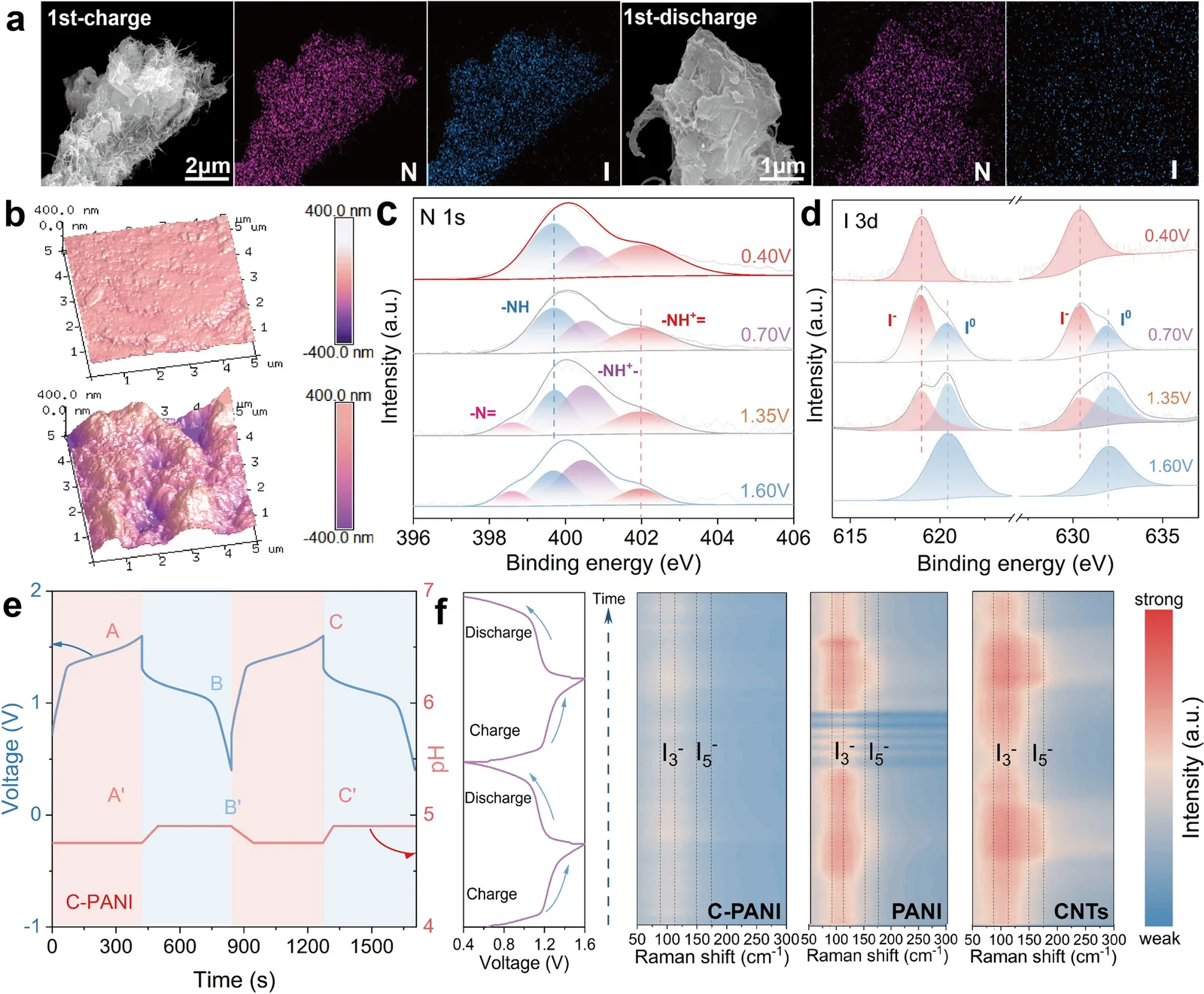
Reaction mechanism of assembled Zn–I_2_ batteries with C-PANI cathode in 2 M Zn(OTF)_2_/0.3 M NH_4_I electrolyte. **a** SEM image of the C-PANI electrode in different charged and discharged states. **b** Three-dimensional AFM images of cycled Zn anode matched with C-PANI and PANI. Ex situ spectra of high-resolution XPS **c** N 1*s* and **d** I 3*d* for the C-PANI at different working states. **e** In situ pH changes of aqueous Zn–I_2_ batteries at the C-PANI side. **f** In situ Raman spectra for the C-PANI, PANI, and carboxyl-CNTs cathodes during cycling

To monitor the microstructure evolution and investigate the redox chemistry of the C-PANI electrode during battery cycling, various *in/*ex situ characterizations were performed. Figures 4a and S25 show the ex situ SEM and corresponding EDS mapping images of the C-PANI electrode after the first two cycles in 2 M Zn(OTF)_2_/0.3 M NH_4_I electrolyte. During the charge/discharge process, the iodine content firstly increased and then decreased in heterostructure C-PANI with C and N elements, indicating the high-efficient iodine conversion induced by redox-catalytic C-PANI for high-performance Zn-I_2_ batteries. Moreover, two- and three-dimensional atomic force microscope (AFM) images of the cycled Zn anodes matched with C-PANI, PANI, and carboxyl-CNTs cathodes, respectively, are displayed in Figs. 4b and S26. Compared with the other two anodes, the smoother surface of the cycled Zn anode coupled with C-PANI suggested the suppressed Zn corrosion caused by the inhibited polyiodide shuttling of C-PANI (Fig. S27). According to previous reports, the preferred orientation of Zn (002) has vital importance for the uniform Zn deposition in durable Zn batteries. As shown in the XRD patterns and SEM images, the cycled Zn anodes matched with C-PANI cathode exhibit the highest I_(002)_/I_(101)_ intensity ratio of 0.66 compared to the other two cycled Zn anodes coupled with PANI (0.20) and carboxyl-CNTs (0.08), which is in consistent with the enhanced stability and uniform Zn deposition for long-cycle Zn-I_2_ batteries (Fig. S28) [23]. Additionally, ex situ N 1*s* and I 3*d* XPS spectra vividly revealed the robust catalytic conversion ability of C-PANI for the reversible I^−^/I^0^ reaction during battery cycling. Specifically, the I^0^ peaks (620.5/632 eV) were well fitted in I 3*d* XPS spectra at the fully charged 1.6 V, suggesting the high-efficient oxidation reaction of I^−^  → I^0^ during the charge process. Conversely, the mere presence of I^−^ peaks located at 619 and 630.4 eV indicated the reduction reaction of I^0^ → I^−^ during the following discharge process (Fig. 4d). Such results suggested the strong catalytic ability of C-PANI for the effective conversion of I^−^/I^0^ without the formation of polyiodide ions. In contrast, the formation of I_3_^−^ and residual I^0^ were observed in the XPS spectra of bare PANI cathode at 0.4 and 1.6 V, respectively, thus inducing lower capacity and inferior cycling stability of Zn-I_2_ batteries (Fig. S29) [27]. Compared with bare PANI, C-PANI generated more positively charged -NH^+^ = /-NH⁺- sites at the fully discharged state, providing the strong chemisorption with the iodine species for efficient I^−^/I^0^ conversion (Figs. 4c and S30). Moreover, the reversible redox reaction of -NH-/-N = endows the catalytic C-PANI cathode with the co-regulated “proton-iodine” effect for high-performance Zn-I_2_ batteries with high capacity and excellent stability. To better prove the feasible protonation process, in situ pH tests of the electrolyte were conducted at the cathode side during the first two battery cycles (Fig. S31). Specifically, the sudden increase and decrease in pH values at the fully discharged and charged states were induced by the proton uptake and removal within the C-PANI cathode, revealing the “proton-iodine” co-regulation based on the = N − /-NH- and I^−^/I^0^ reactions (Figs. 4d, e and S32). In situ Raman spectra of the three cathodes were collected for the in-depth exploration of effective iodine conversion catalyzed by redox-active C-PANI (Fig. 4f). During the first two cycles, merely a few I_3_^−^ ions were observed in the C-PANI cathode, while obvious I_3_^−^ and I_5_^−^ signals were measured within PANI and carboxyl-CNTs cathodes, especially at the redox platform ~ 1.2 V, which is consistent with ex situ XPS results [23]. Therefore, the synergistic “proton-iodine” regulation endows catalytic-active C-PANI with high-efficient I^−^/I^0^ chemistry along with inhibited polyiodide shuttling induced by -NH^+^-/-NH^+^ = (iodine adsorption) and -N = (proton storage) groups, thus achieving high-capacity and robust Zn-I_2_ batteries [10].

### Related DFT Calculation and Practical Performance of Zn–I_2_ Pouch Cell

**Fig. 5:**
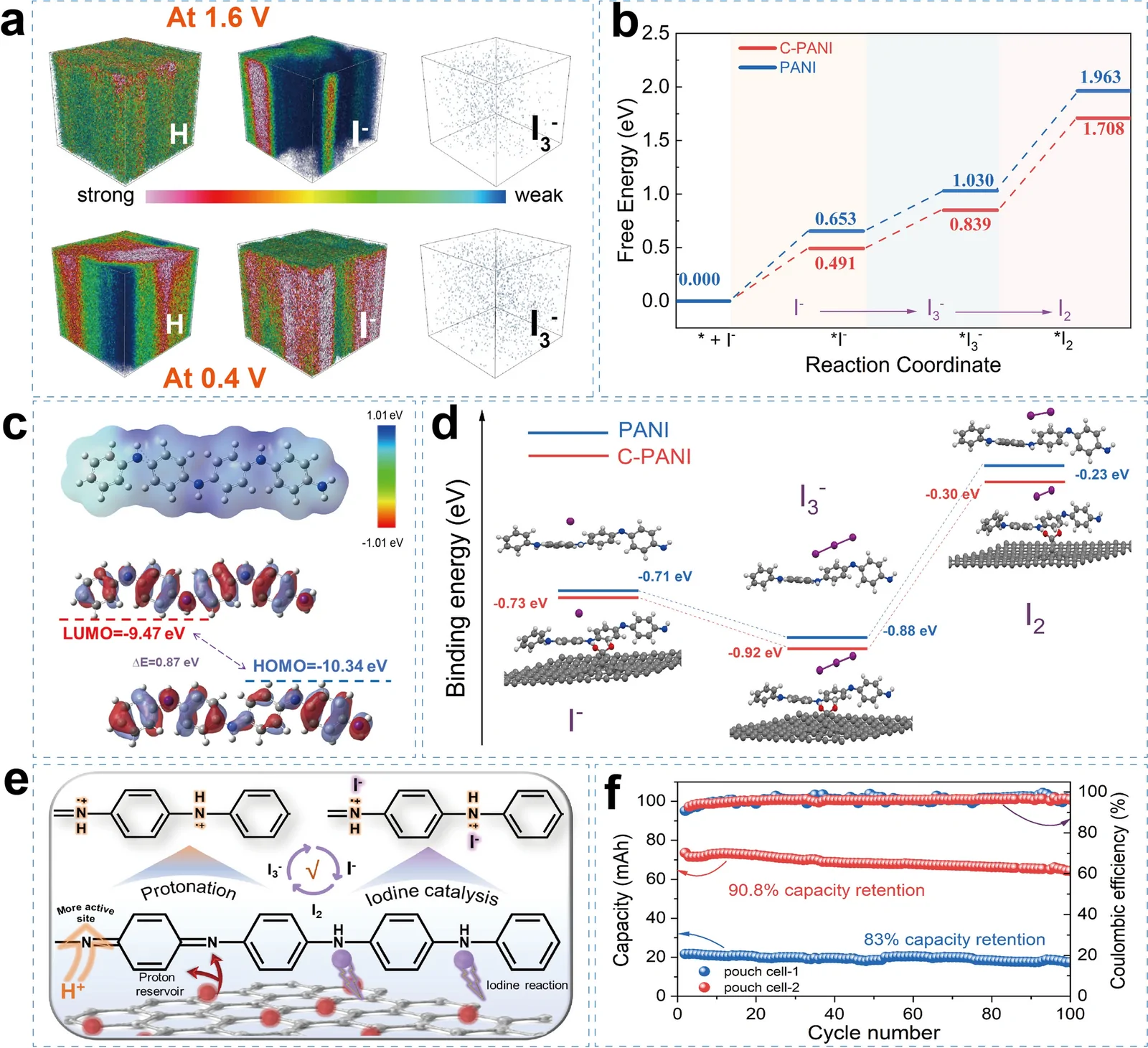
Related DFT simulation and Zn-I_2_ pouch cell performance based on C-PANI cathode. **a** TOF–SIMS depth-scan image of the C-PANI electrode at the fully charged and discharged states, respectively. **b** Gibbs free energy diagram of the I^−^ ion oxidation reaction of the C-PANI and PANI. **c** Upside: Isosurface map of ESP for PANI (isovalue = 0.05 au). Blue and red isosurfaces correspond to positive and negative parts of ESP, respectively. Downside: HOMO and LUMO of discharged PANI. **d** Calculated binding energy of iodine species (I^−^, I_2_, and I_3_^−^) on PANI and C-PANI. **e** Energy storage mechanism of iodine conversion chemistry based on C-PANI cathode. **f** Cycling of two different sized Zn–I_2_ pouch cells

To further clarify the redox-catalytic ability of C-PANI, the distribution of H^+^, I⁻, I_3_⁻, and I_5_⁻ species within the C-PANI electrode was analyzed by employing time-of-flight secondary ion mass spectrometry (TOF–SIMS) during battery cycling. Compared with the discharged state (0.4 V), C-PANI electrode exhibits much lower concentration of H^+^ and I⁻ ions at the fully charged state (1.6 V), proving the efficient co-storage of H^+^ and I⁻ ions (Fig. 5a). Notably, the polyiodide ions including I_3_⁻ and I_5_⁻ were rarely measured within the C-PANI electrode at both 1.6 and 0.4 V (Figs. S33 and S34) [43]. This observation is consistent with the results from in situ Raman and ex situ XPS analyses, reconfirming the high-efficient I⁻/I^0^ with efficient polyiodide confinement compared to the previous reported I⁻/I_3_⁻ redox for electrolytic Zn-I_2_ batteries [22–24]. Figure 5c depicts the electrostatic potential (ESP) distribution of protonated PANI to confirm the proton uptake sites (-N =) and iodine anchor sites (-NH^+^-/-NH^+^ =). Moreover, the energy gap of C-PANI (0.87 eV) is smaller than that of PANI (2.29 eV) between its highest occupied molecular orbital (HOMO) and lowest unoccupied molecular orbital (LUMO), which facilitates efficient electron transfer, thereby accelerating iodine redox kinetics (Fig. S35) [44, 45]. The Gibbs free energies of the oxidation pathway (I^−^  → I_3_^−^ → I^0^) were calculated within the two catalytic cathodes, as depicted in Fig. 5b [46]. Notably, a lower ΔG value typically indicates a higher degree of spontaneity and a faster reaction rate for the iodine conversion reaction. Specifically, the Gibbs free energy barrier for iodine conversion at the C-PANI cathode (0.869 eV) is significantly lower than that at the PANI cathode (0.933 eV), further demonstrating the enhanced I⁻/I_2_ conversion efficiency caused by the “proton-iodine” co-regulation effect. To stress the strong iodine fixation ability, density functional theory (DFT) calculations was employed on the binding energy of iodine species onto C-PANI and PANI hosts, respectively (Fig. 5d). As a result, the binding energy of all iodine species on C-PANI is lower than that on bare PANI electrode, especially for I_3_^−^, suggesting the significant role of C-PANI on anchoring multiple iodine ions. We further utilized a schematic diagram to illustrate the boosted I^−^/I^0^ conversion and effective polyiodide constraint ability by redox-catalytic C-PANI cathode with protonated -NH^+^- and -NH^+^ = sites, revealing the “proton-iodine” interaction for enhanced capacity and stability of electrolytic Zn-I_2_ batteries (Fig. 5e) [47]. The outstanding electrochemical performance of the C-PANI electrode underscores its practical application potential through the two Zn-I_2_ pouch cells based on C-PANI cathodes and NH_4_I containing electrolyte (Figs. S36 and S37). As shown in Fig. 5f, these two pouch batteries exhibit capacities of 21 and 72 mAh, respectively, while retaining 83% and 90.8% of their initial capacity after 100 charge–discharge cycles. Figure S38 displays the 100th GCD profiles of the two punch cells, proving the robust catalytic property of C-PANI on direct I^−^/I^0^ reaction. Through the battery performance comparison, C-PANI cathode exhibits superior cycling stability and capacity compared to reported electrolytic Zn-I_2_ pouch cells (Fig. S39 and Table S2) [29, 36, 48–50]. Furthermore, we conducted device demonstrations of a light-emitting diode (LED) board powered by the pouch cells, thereby confirming their practical applicability (Fig. S40) [51]. To better assess practical feasibility, we calculated the negative-to-positive (N/P) ratio and energy density of the pouch cell (Table S3) [52, 53]. Therefore, redox-catalytic C-PANI cathode provides effective “proton-iodine” co-regulation for enhanced I^−^/I^0^ conversion kinetics along with negligible polyiodide shuttling for high-rate and robust Zn-I_2_ batteries [54, 55].

## Conclusions

In conclusion, we explored neuron-inspired C-PANI as the redox-catalytic cathode featuring “proton-iodine” co-storage for electrolytic Zn-I_2_ batteries with a high capacity of 423 mAh g^−1^ at 1 A g^−1^ and outstanding capacity retention (223 mAh g^−1^) after 40,000 cycles at 20 A g^−1^. Interestingly, the incorporation of carboxyl-CNTs and PANI not only facilitates the formation of electron/charge transfer tunnels within three-dimensional C-PANI networks but also enhances the physicochemical confinement of iodine species for inhibited polyiodide shuttling. Various *in*/ex situ characterizations coupled with DFT calculations revealed that the catalytic C-PANI cathode exhibited the high-efficient I^−^/I^0^ conversion chemistry along with high Coulombic efficiency during long-term battery working. More importantly, redox-catalytic C-PANI with proton self-limiting property can effectively boost the proton uptake chemistry during the discharge process, providing more protonated -NH⁺- and -NH⁺ = sites to anchor and suppress polyiodide shuttling. Besides, C-PANI significantly decreases the binding energy with iodine species, particularly I^−^ and I_3_^−^ ions, promoting the thorough catalytic conversion of I^−^  → I^0^ rather than most reported I^−^  → I_3_^−^ redox in electrolytic cathodes. Additionally, the assembled pouch cell with the mass-prepared C-PANI cathode achieves a stable ~ 60 mAh after 100 cycles, providing novel insights for effective organic catalysts based on the synergistic “proton-iodine” effect.

## Supplementary Information

Below is the link to the electronic supplementary material.

Supplementary file1 (DOCX 56097 KB)
