# Supplementary material for: “Proton-Iodine” Regulation of Protonated Polyaniline Catalyst for High-Performance Electrolytic Zn-I2 Batteries
Source: Nanomicro Lett. 2025 Nov 1;18:79. doi: 10.1007/s40820-025-01928-5 (PMC12579043; doi:10.1007/s40820-025-01928-5)
Supplement: Supplementary file 1 — Supplementary file1 (DOCX 56097 KB) [file 40820_2025_1928_MOESM1_ESM.docx]

Supporting Information for

**“Proton-Iodine” Regulation of Protonated Polyaniline Catalyst for High-Performance Electrolytic Zn-I_2_ Batteries**

Mengyao Liu^1^, Kovan Khasraw Abdalla^1^, Meng Xu^1^, Xueqian Li^2^, Runze Wang^1^, Qi Li^1^, Xiaoru Zhang^1^, Yanan Lv^1^, Yueyang Wang^1^, Xiaoming Sun^1^, Yi Zhao^1,3*^

^1^ State Key Laboratory of Chemical Resource Engineering, Beijing Advanced Innovation Center for Soft Matter Science and Engineering, College of Chemistry, Beijing University of Chemical Technology, Beijing 100029, P. R. China

^2^ Beijing Key Laboratory of Environmental Science and Engineering, School of Materials Science and Engineering, Beijing Institute of Technology, Beijing 100081, P. R. China

^3^ Shandong Key Laboratory of Advanced Chemical Energy Storage and Intelligent Safety, Advanced Technology Research Institute, Beijing Institute of Technology, Jinan 250300, P. R. China

*Corresponding author. E-mail: [zybattery@buct.edu.cn](mailto:zybattery@buct.edu.cn) (Yi Zhao)

**S1 Experimental Section**

**S1.1 Materials**

Polyaniline (PANI) with 98% purity, carboxyl-carbon nanotubes (carboxyl-CNTs), zinc trifluoromethanesulfonate (Zn(OTF)_2_), ammonium iodide (NH_4_I), N-methyl-2-pyrrolidone (NMP), and all other chemicals were purchased from Alfa Aeser Pvt. Ltd and used without further purification. Carbon cloth (WOS1009, CeTech. Co.) exhibits enhanced hydrophilicity following treatment with a plasma testing device.

**S1.2 Preparation of C-PANI cathode materials**

The synthesis of the inorganic/organic cathode host was initiated at room temperature by meticulously blending polyaniline (PANI), carboxylated carbon nanotubes (cCNTs) as the conductive agent, and a PVDF binder in a precise mass ratio of 6:3:1. N-methyl-2-pyrrolidone (NMP) were employed as the solvent medium to ensure a homogeneous dispersion of the components. The resulting mixture was then uniformly coated onto a carbon cloth substrate and compressed to achieve a consistent layer. Following this, the coated substrate was subjected to a controlled drying process at 60 °C in a vacuum oven, maintained overnight to ensure complete solvent evaporation and optimal adhesion of the composite materials. The fabricated electrodes demonstrated a uniform PANI mass loading of approximately 1 ⁓ 2 mg cm⁻^2^, ensuring reproducibility and reliability in subsequent electrochemical evaluations. For comparative analysis, a control sample comprising only carboxyl-CNTs and PANI were prepared under identical conditions to isolate the effects of the composite structure. Throughout the synthesis process, stringent protocols were adhered to, guaranteeing consistent electrode homogeneity and robust compatibility between the inorganic and organic components.

**S1.3 Materials characterization**

Powder X-ray diffraction (XRD) analysis was carried out on Rigaku Ultima IV with the Cu Kα radiation source. The samples were scanned at a 2θ range of 5 to 80° with a scan speed of 5° min⁻^1^. Surface chemical composition and element valence were determined with X-ray photoelectron spectroscopy (XPS, ESCALAB 250 Thermo Fisher Scientific) and a Raman spectrometer with an Ar laser excitation source at 532 nm (LabRAM Aramis, HORIBA Jobin Yvon), respectively. Specific surface area and pore-size distribution of cathode materials were measured on a Micromeritics ASAP 2020 adsorption analyzer based on the Brunauer−Emmert−Teller (BET) method.

**S1.4 Electrochemical measurements**

The fabricated C-PANI electrode is employed as the cathode of the battery, with zinc foil serving as the anode and glass fiber acting as the diaphragm. The electrolyte consists of an aqueous solution of 2 M Zn(OTF)_2_ and 0.3 M NH_4_I. The battery is assembled within the CR2032 coin-type cells. Herein, the loading of the active material on the cathode sheet ranges from 1.0 to 2 mg cm⁻^2^, and approximately 140 μL of the electrolyte is added to each single button battery. The mass loading of active substances in the pouch cell were 10.72 mg cm⁻^2^,17.88 mg cm⁻^2^, respectively. Their sizes are 4.55×2.7 and 4.7×6.3 cm^2^, respectively. The electrolyte is 2 M Zn(OTF)_2_ and 0.3 M NH_4_I, and the added amounts are 5ml and 10ml, respectively. Cycle voltametry (CV) and electrochemical impedance spectroscopy (EIS) spectra were obtained using a CorrTest CS2350H electrochemical workstation (Wuhan CorrTest Instrument Corp., Ltd. China). The galvanostatic charge–discharge (GCD) and cycling performance were carried out with a NEWARE Battery Test System (Neware, Shenzhen, China).

**S1.5 Theoretical calculation**

The molecular structure, energy levels (HOMO and LUMO), and molecular electrostatic potential (ESP) of PANI were computed using the Gauss 09 software package. At the B3LYP level of theory, the molecular geometries of both PANI and C-PANI were fully optimized using dispersion-corrected density functional theory (DFT-D3) with the 6-31G(d) basis set [S1, S2]. All the DFT calculations were conducted based on the Vienna Ab initio Simulation Package (VASP) [S3, S4]. The exchange-correlation potential was described by the Perdew−Burke−Ernzerhof (PBE) generalized gradient approach (GGA) [S5]. The electron-ion interactions were accounted by the projector augmented wave (PAW) [S6]. All DFT calculations were performed with a cut-off energy of 400 eV, and the Brillouin zone was sampled with the gamma (Γ) k-point. The energy and force convergence criteria of the self-consistent iteration were set to 10⁻^4^ eV and 0.02 eV Å⁻^1^, respectively. The DFT-D3 method was used to describe van der Waals (vdW) interactions [S7].

The adsorption energies (∆*E*) are calculated by the following formula:

∆E = E_*A_–E_A_ –E_Sub_ (S1)

where *E*_C-PANI_ and *E*_*C-PANI_ represent the energies before and after the adsorption of A molecule on the substrate, respectively. And *E*_sub_ is the energy of a clean surface.

The Gibbs free energy changes (ΔG) of the reaction are calculated using the following formula:

∆G = ∆E + ∆ZPE - T∆S (S2)

where ΔE is the difference of electron energies calculated by DFT; ΔZPE and ΔS are the changes of zero-point energy and entropy, respectively, which are obtained from vibrational frequencies. T is the temperature (298.15 K).

**S1.6 Calculation of Ion Diffusion Coefficient (D) from EIS Tests**

Based on the low-frequency spectrum of EIS, the ion diffusion coefficient can be calculated by the following equation [S8]:

$Z^{'}=R_{\text{s}}+R_{ct}+\sigma\omega^{-0.5}$ (S3)

$D=\frac{R^{2}T^{2}}{2A^{2}n^{4}F^{4}C^{2}\sigma^{2}}$ (S4)

where ω, A, n, F, C, R, and T denote the Warburg factor, electrode area, number of electrons involved in each electrochemical reaction, Faraday constant (96,500 C∙mol^-1^), molar concentration of ions, gas constant (8.314 J mol⁻^1^∙K⁻^1^), and absolute temperature (K), respectively. The fundamental wave component Z' encompasses bulk resistance (Rs), charge transfer resistance (Rct), and mass transfer resistance. The values of Rs and Rct are derived from equivalent circuit fitting procedures.

**S1.7 Calculation of Ion Diffusion Coefficient (D)**

After the assembled battery is allowed to rest, it undergoes 20 cycles of discharge/charge at a current density of 0.5 A g⁻^1^. Subsequently, the constant current Intermittent Titration Technique (GITT) is employed to ensure that the system reaches a stable state. The battery is then subjected to a charging protocol involving an initial charge at 2 A∙g⁻^1^ for 6 minutes, followed by an additional 12 minutes of charging to achieve voltage equilibrium. This procedure is repeated throughout the subsequent charging/discharging cycles. Based on the relationship between potential and time, D can be calculated using the following equation [S9]:

$D=\frac{4}{\pi\tau}\left(\frac{n_{m}V_{m}}{S}\right)^{2}\left(\frac{\Delta E_{s}}{\Delta E_{t}}\right)^{2}$ (S5)

$\tau$ is the relaxation time; 𝑛_𝑚_ is the number of moles, 𝑉_𝑚_ is the molar volume, S is an electrode contact area, $\Delta E_{s}$is a voltage change caused by pulses, and $\Delta E_{t}$is the voltage change of constant current charge and discharge [S10].

**S2 Supplementary Figures and Tables**

**
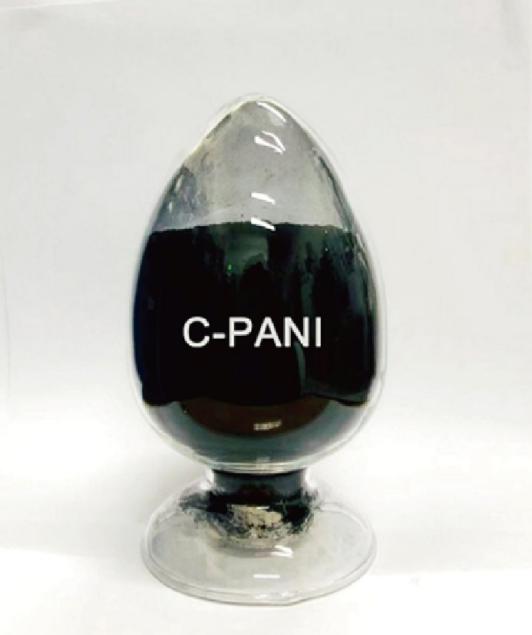
**

**Fig. S1** Large-scale illustration of C-PANI material with a total mass of 30 g


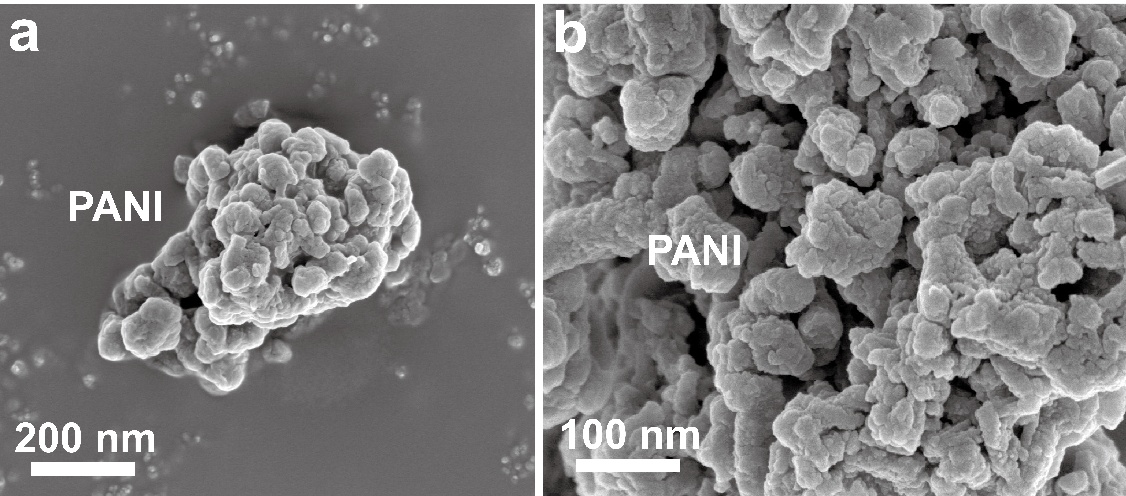


**Fig. S2** SEM images of PANI with nanorod structures at different magnifications

**
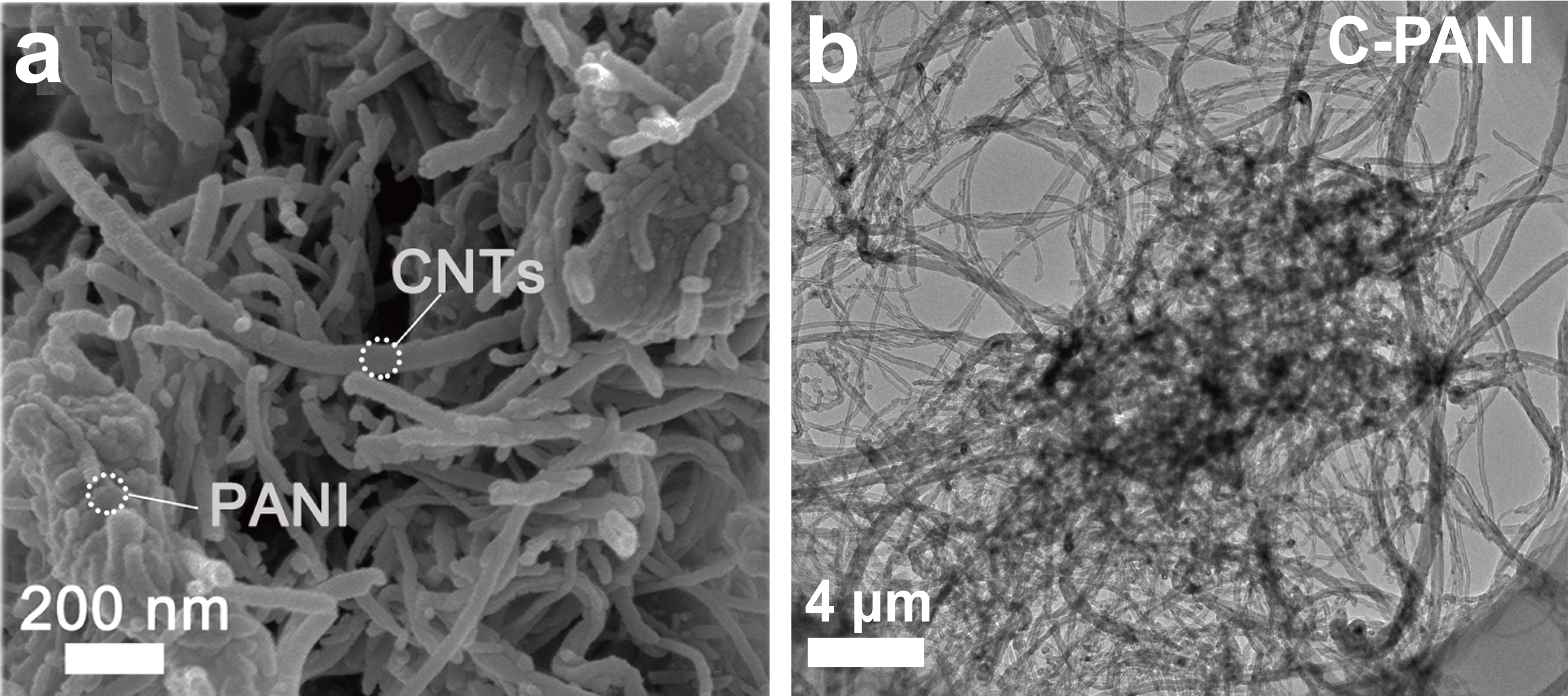
**

**Fig. S3** (**a**) SEM images of C-PANI. (**b**) TEM images of C-PANI


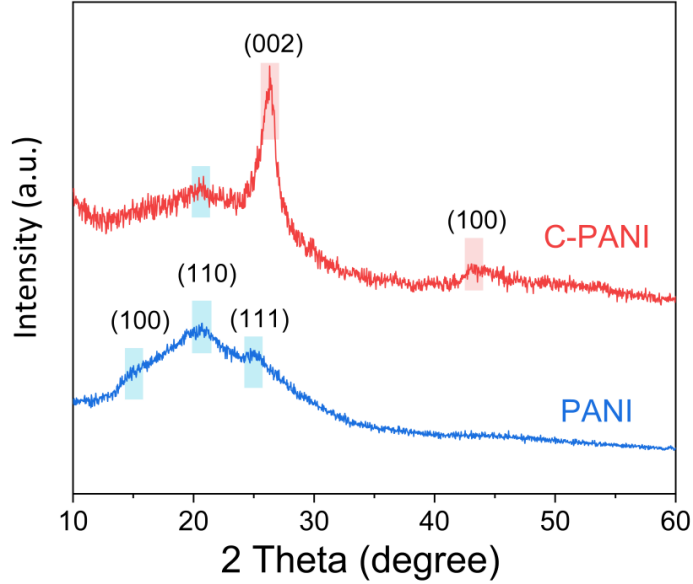


**Fig. S4** XRD patterns of C-PANI and PANI


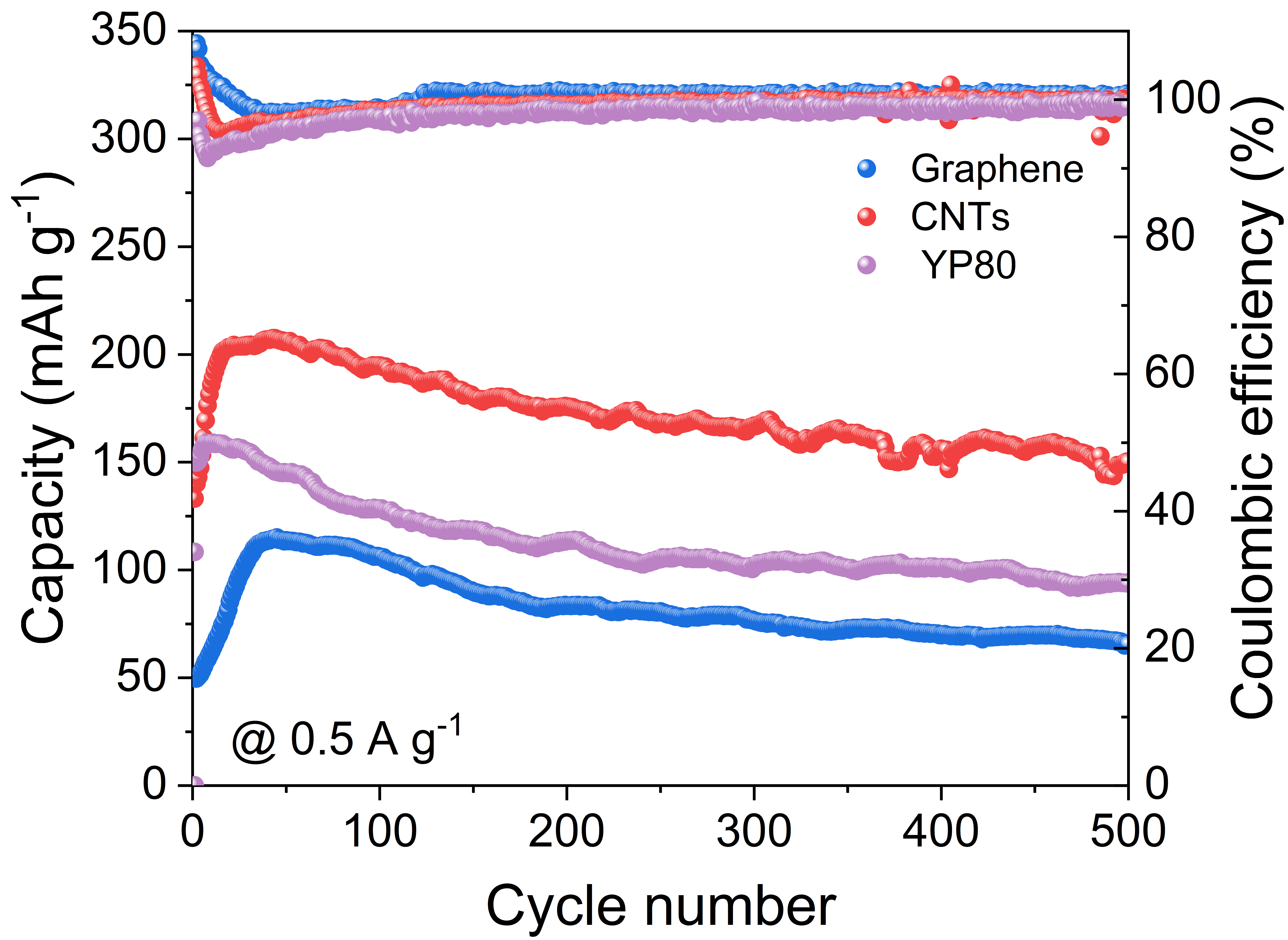


**Fig. S5** The positive electrode materials obtained by the composite of PANI and different kinds of activated carbon are used as the cathode, and three kinds of cyclic performance are obtained in the electrolyte of 2 M Zn(OTF)_2_ at the current density of 0.5 A g^–1^


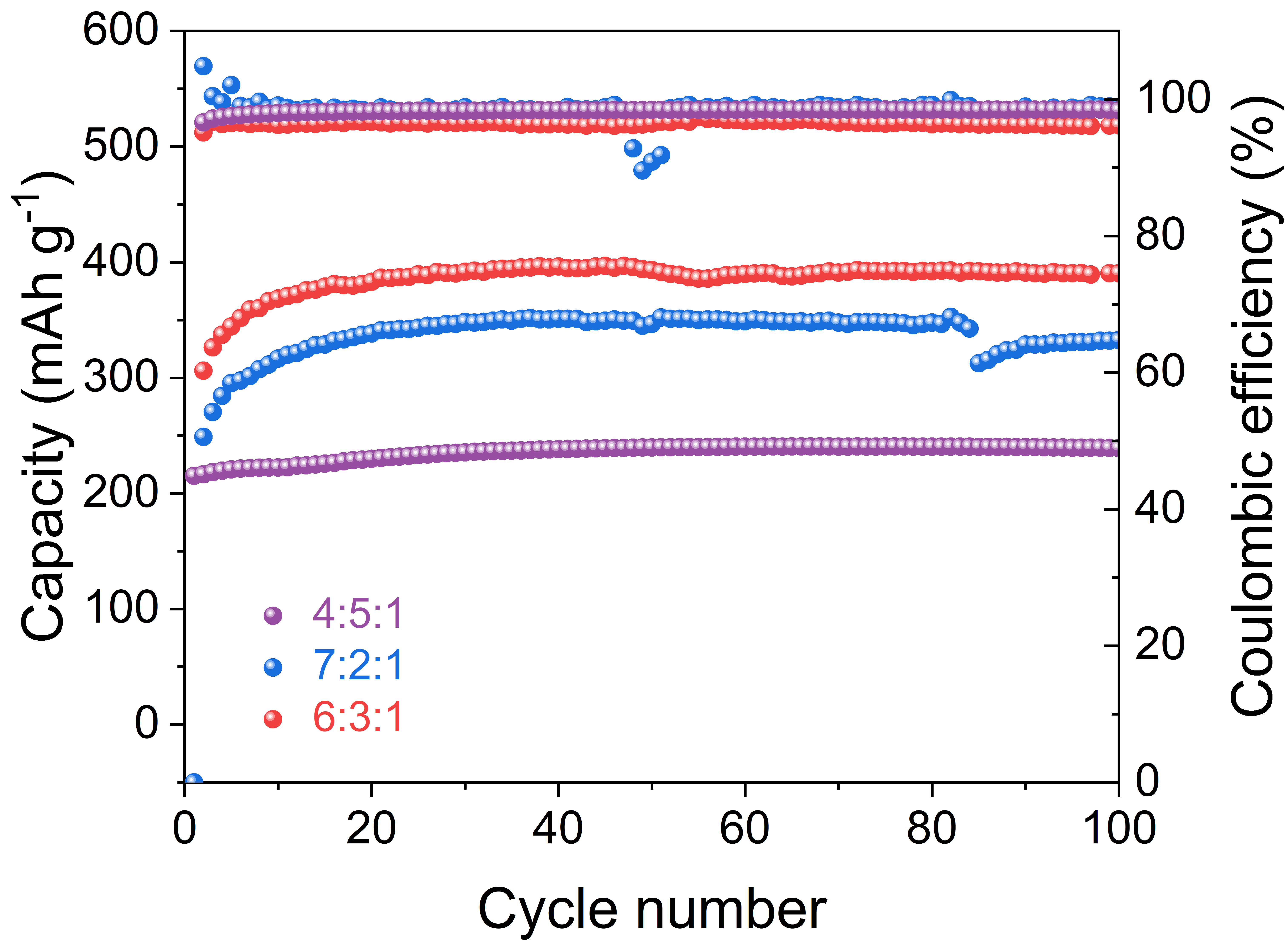


**Fig. S6** The cathodes with different ratio of PANI, carboxyl-CNTs, PVDF, and three kinds of cyclic performance are obtained in the electrolyte of 2 M Zn(OTF)_2_ at the current density of 0.5 A g^–1^





**Fig. S7** The mass ratio of PANI: carboxyl-CNTs: PVDF was chosen as 6:3:1 for C-PANI catalytic cathode, and three kinds of cyclic performance are obtained in the electrolyte of 2 M Zn(OTF)_2_ and different concentrations of ammonium iodide at the current density of 0.5 A g^–1^


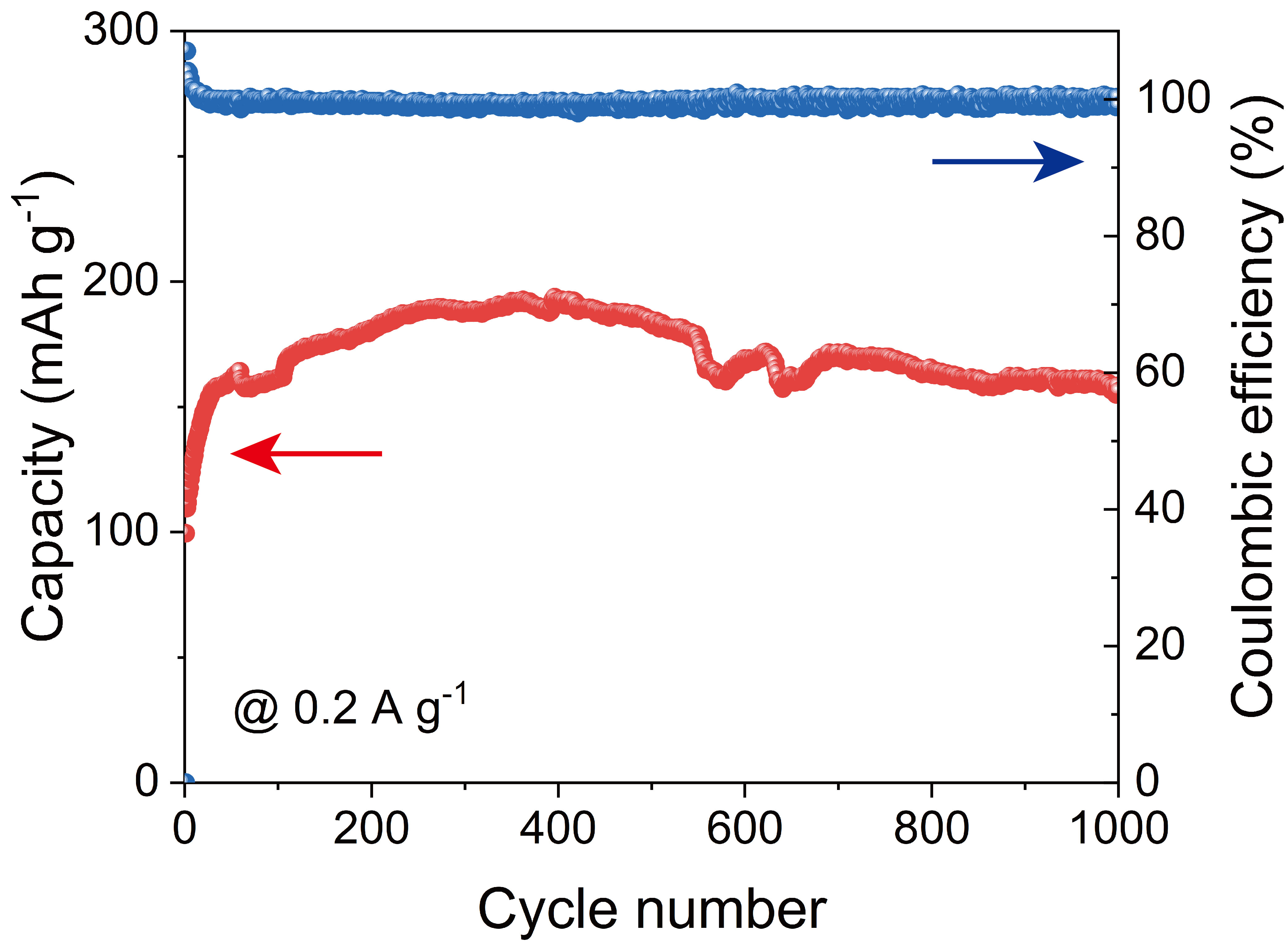


**Fig. S8** Cycling performances of C-PANI at 0.2 A g^–1^ in 2 M Zn(OTF)_2_ electrolyte


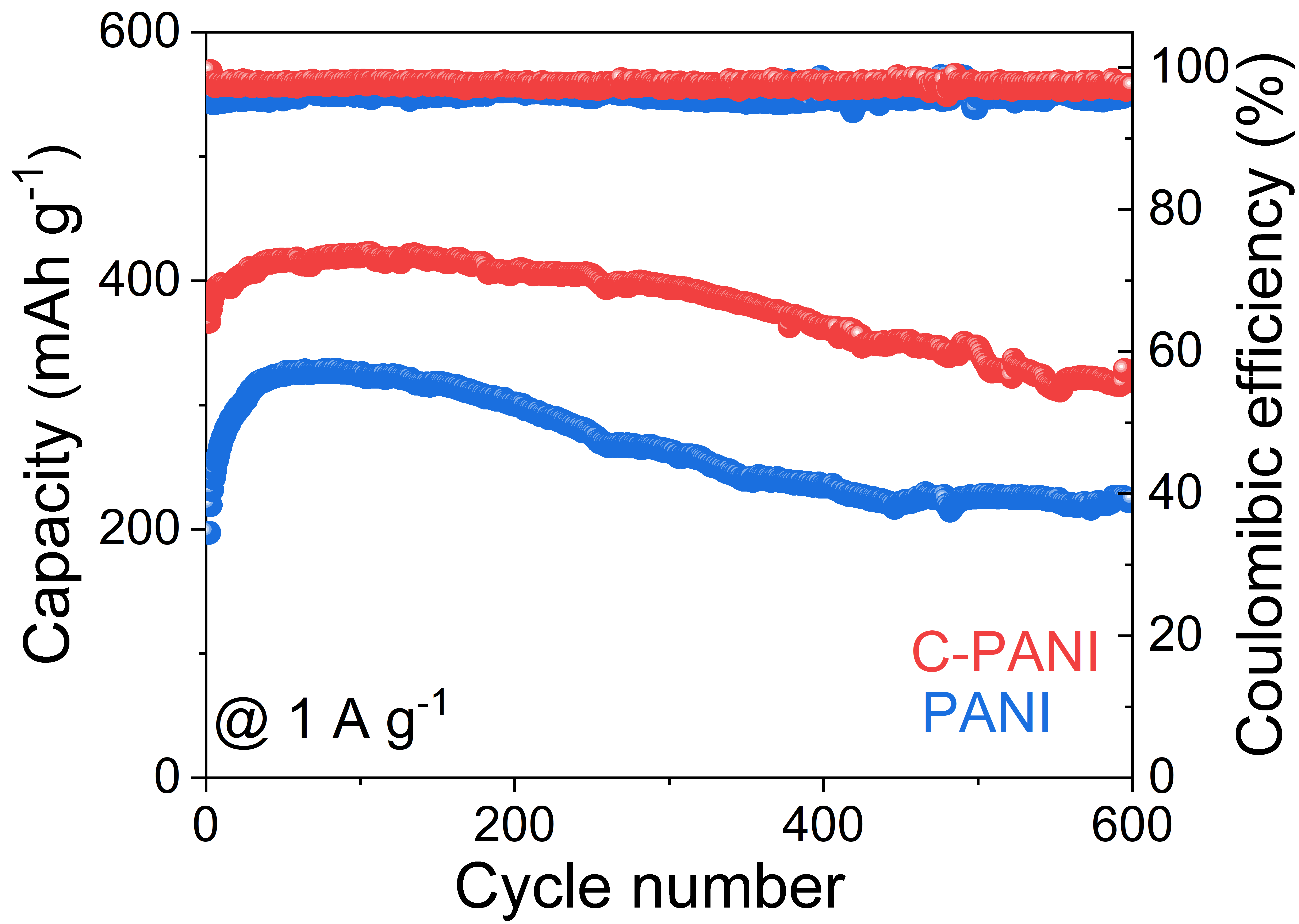


**Fig. S9** Cycling performances of C-PANI and PANI at 1 A g^–1^ in 2 M Zn(OTF)_2_/0.3 M NH_4_I


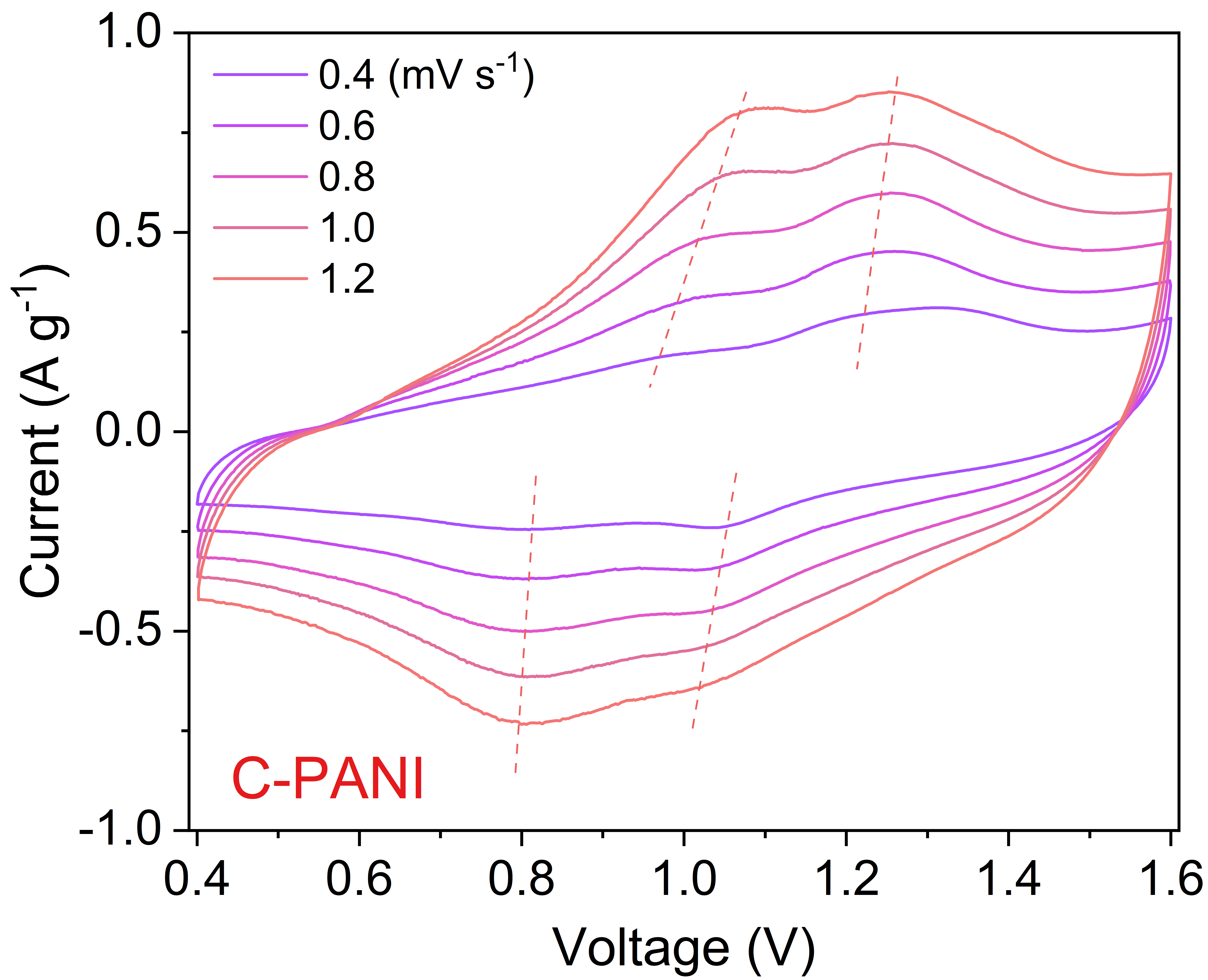


**Fig. S10** The 2^nd^-cycle CV curves of C-PANI at various scan rates in 2 M Zn(OTF)_2_ electrolyte


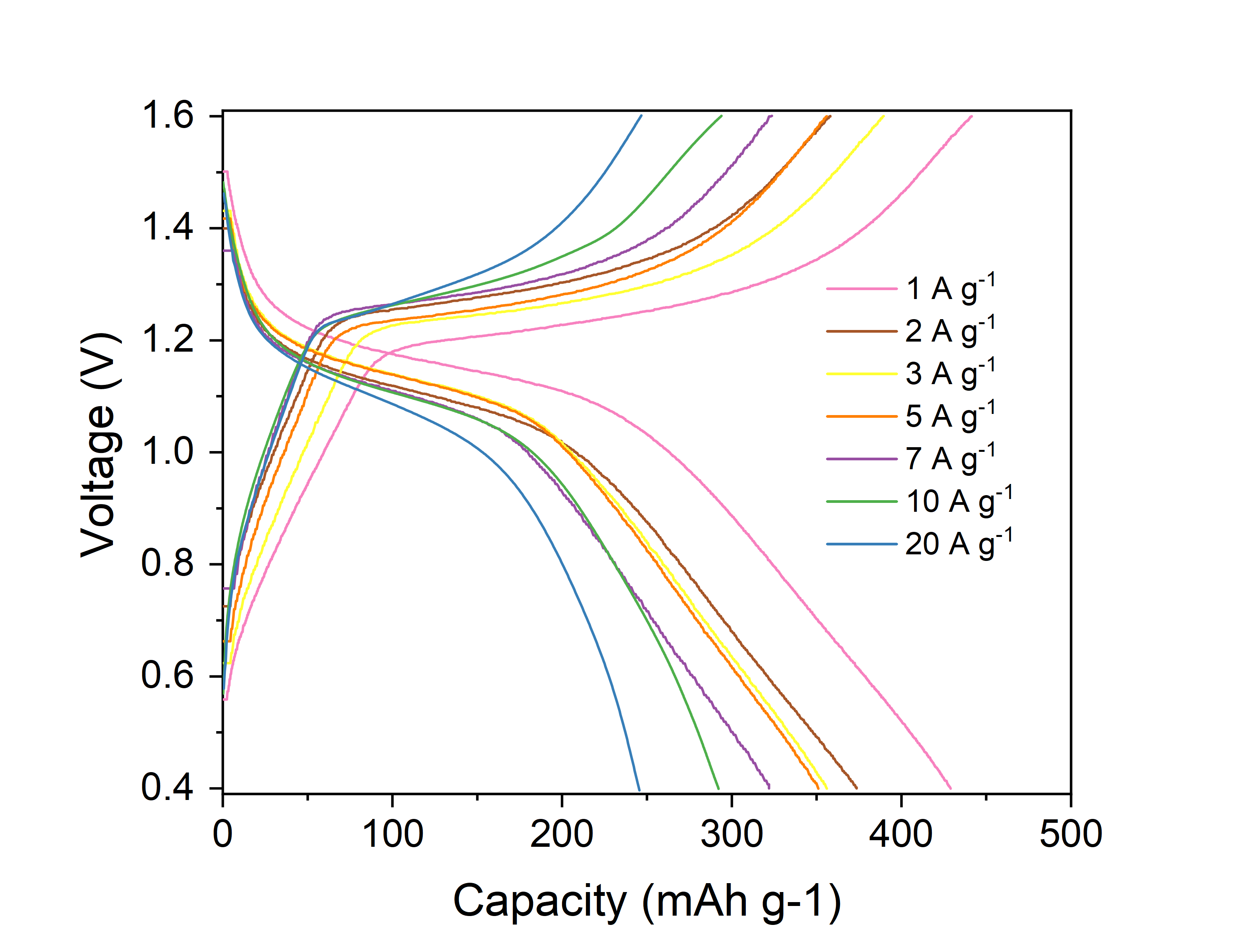


**Fig. S11** GCD curves of C-PANI at 1, 2, 3, 5, 7,10 and 20 A g^–1^ in 2 M Zn(OTF)_2_/ 0.3 M NH_4_I electrolyte

**

**

**Fig. S12** Cycling performances of C-PANI and PANI at 5 A g^–1^ in 2 M Zn(OTF)_2_/0.3 M NH_4_I





**Fig. S13** Cycling performances of C-PANI and PANI at 10 A g^–1^ in 2 M Zn(OTF)_2_/0.3 M NH_4_I

**
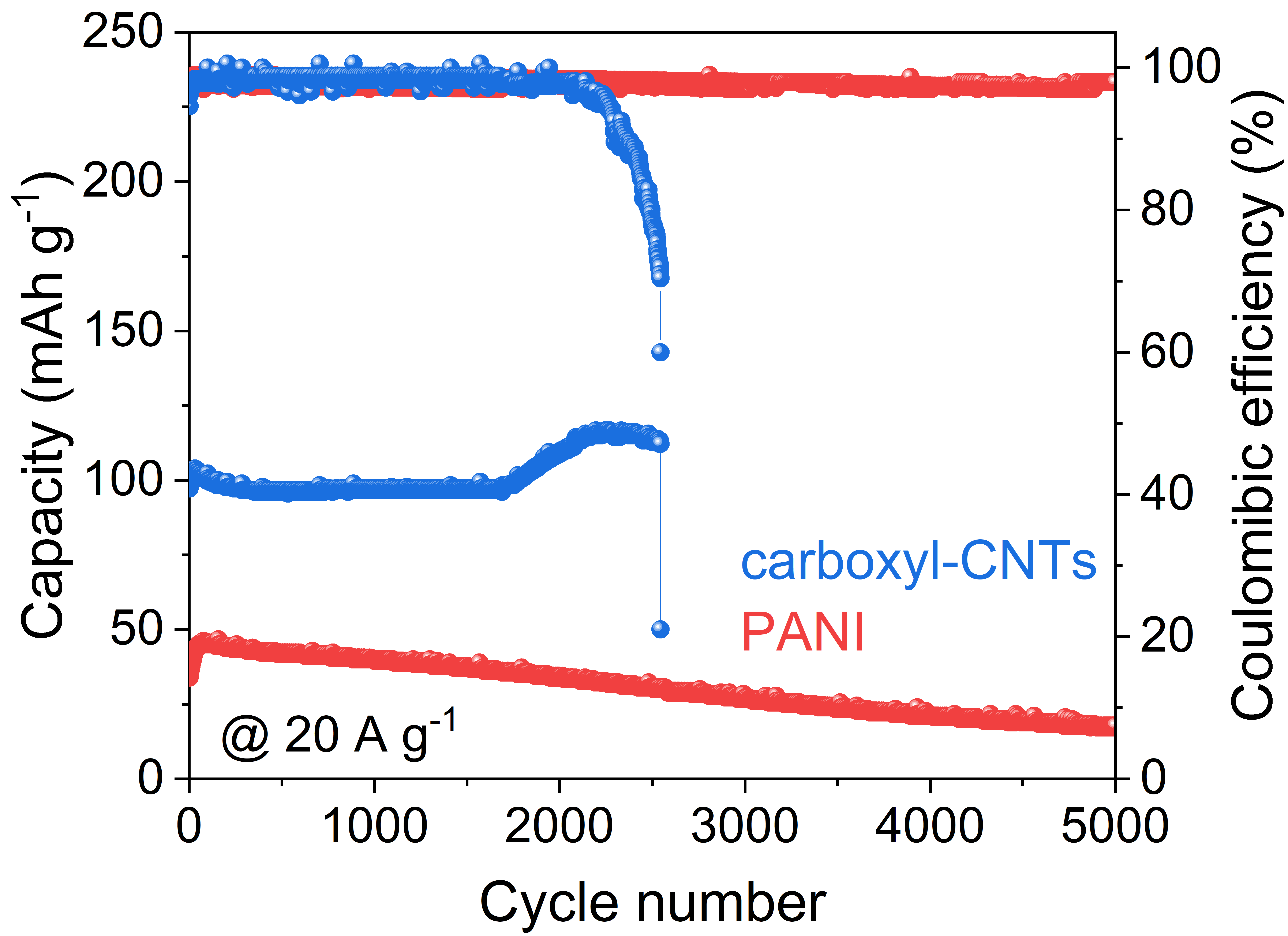
**

**Fig. S14** Cycling performances of PANI and carboxyl-CNTs at 20 A g^–1^ in 2 M Zn(OTF)_2_/0.3 M NH_4_I

**
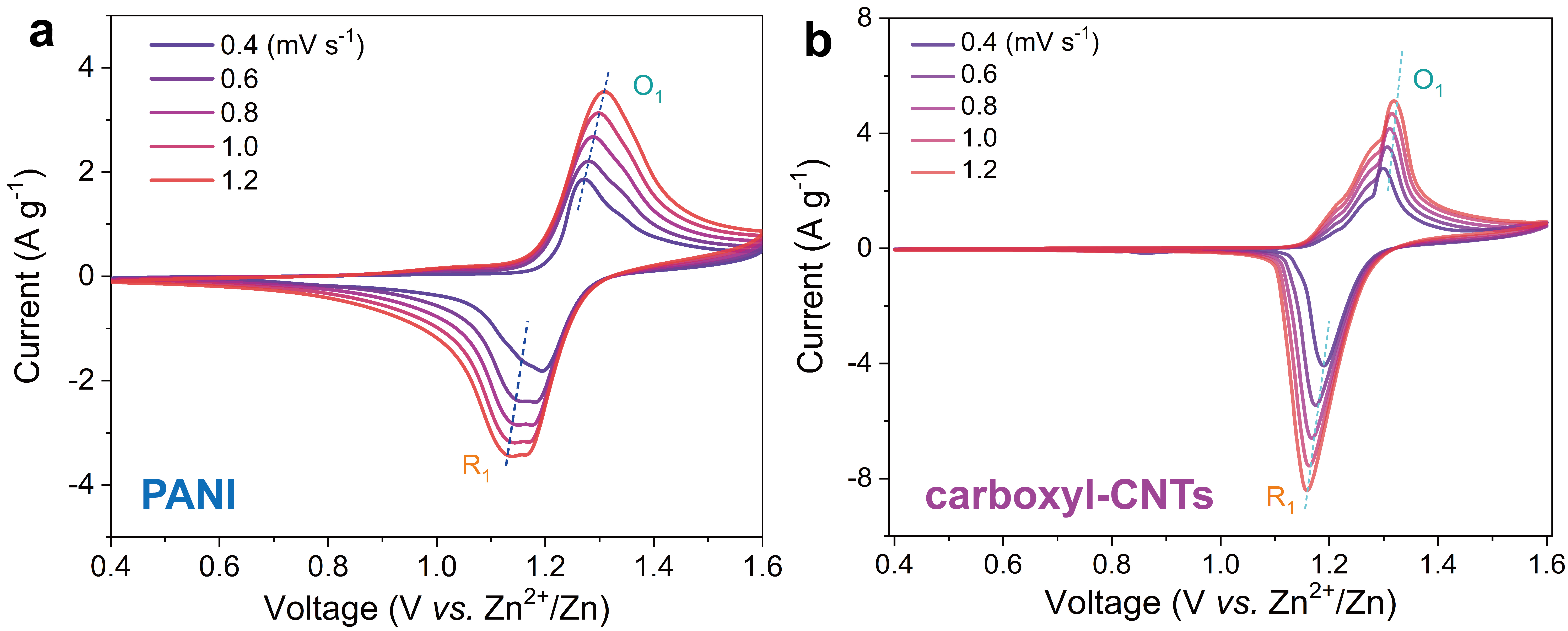
**

**Fig. S15** CV curves of PANI (**a**) and carboxyl-CNTs (**b**) electrode at various scan rates in 2 M Zn(OTF)_2_/0.3 M NH_4_I


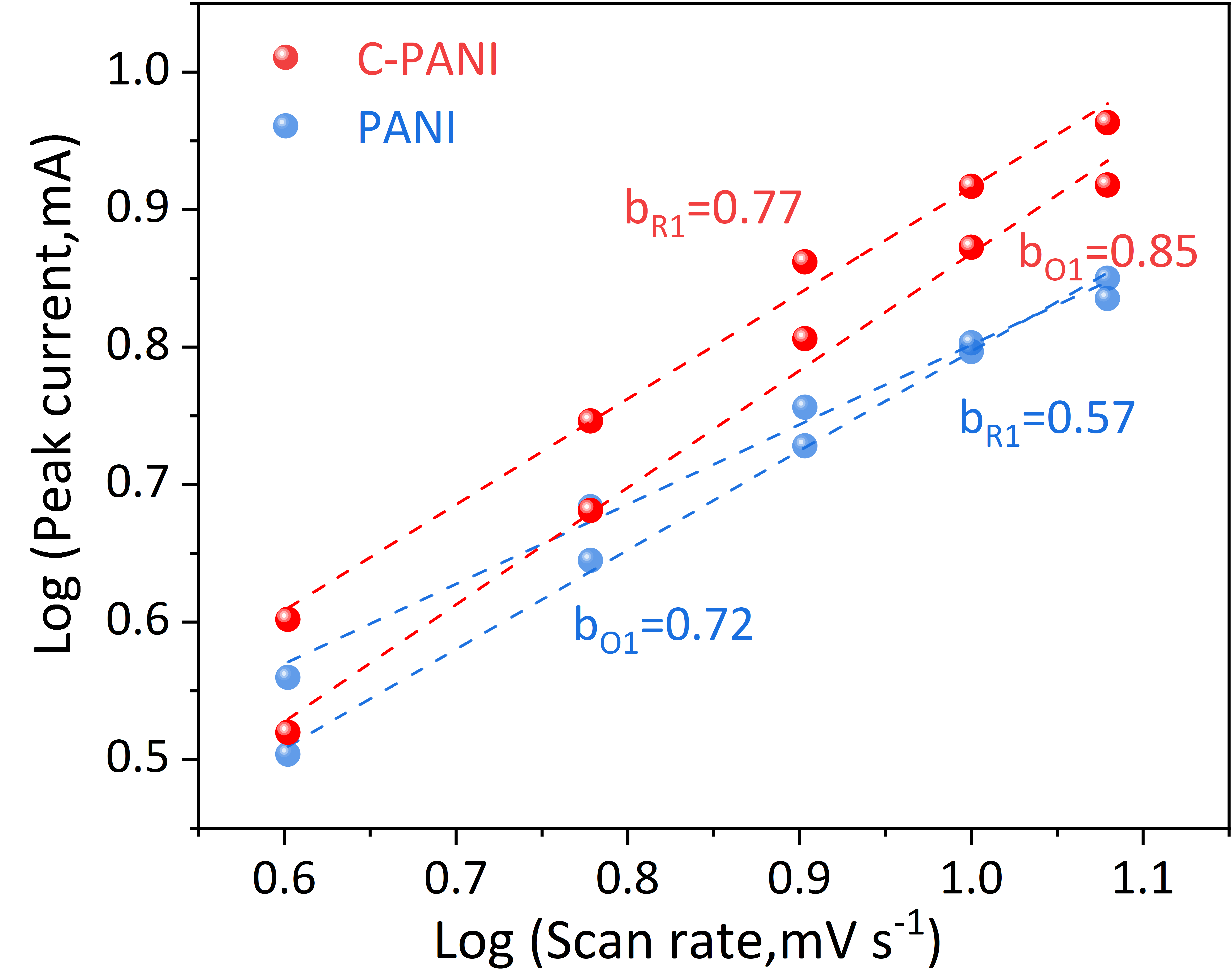


**Fig. S16** The plots of log (current) vs. log (scan rate) of C-PANI (red) and PANI (blue)


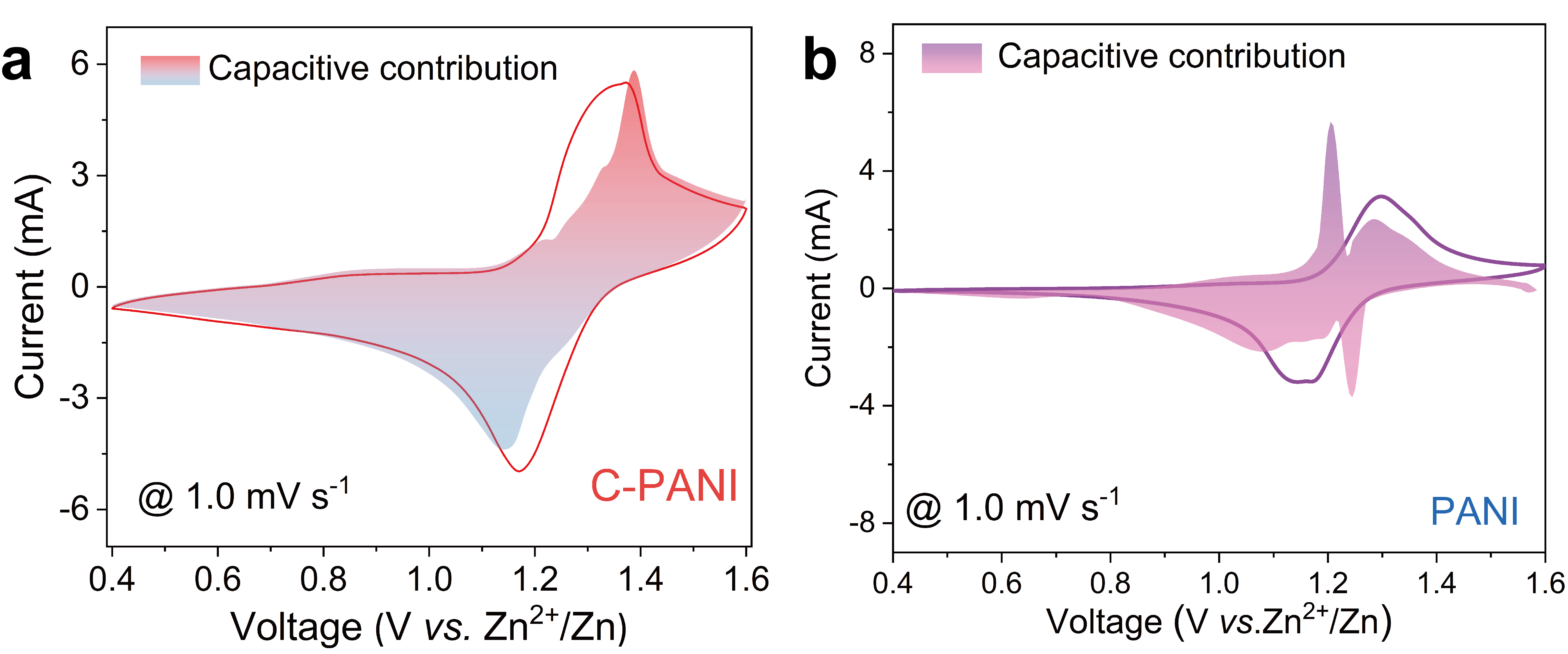


**Fig. S17** Capacitive behaviors contributions of C-PANI (**a**) and PANI (**b**) cathode at 1.0 mV s^–1^


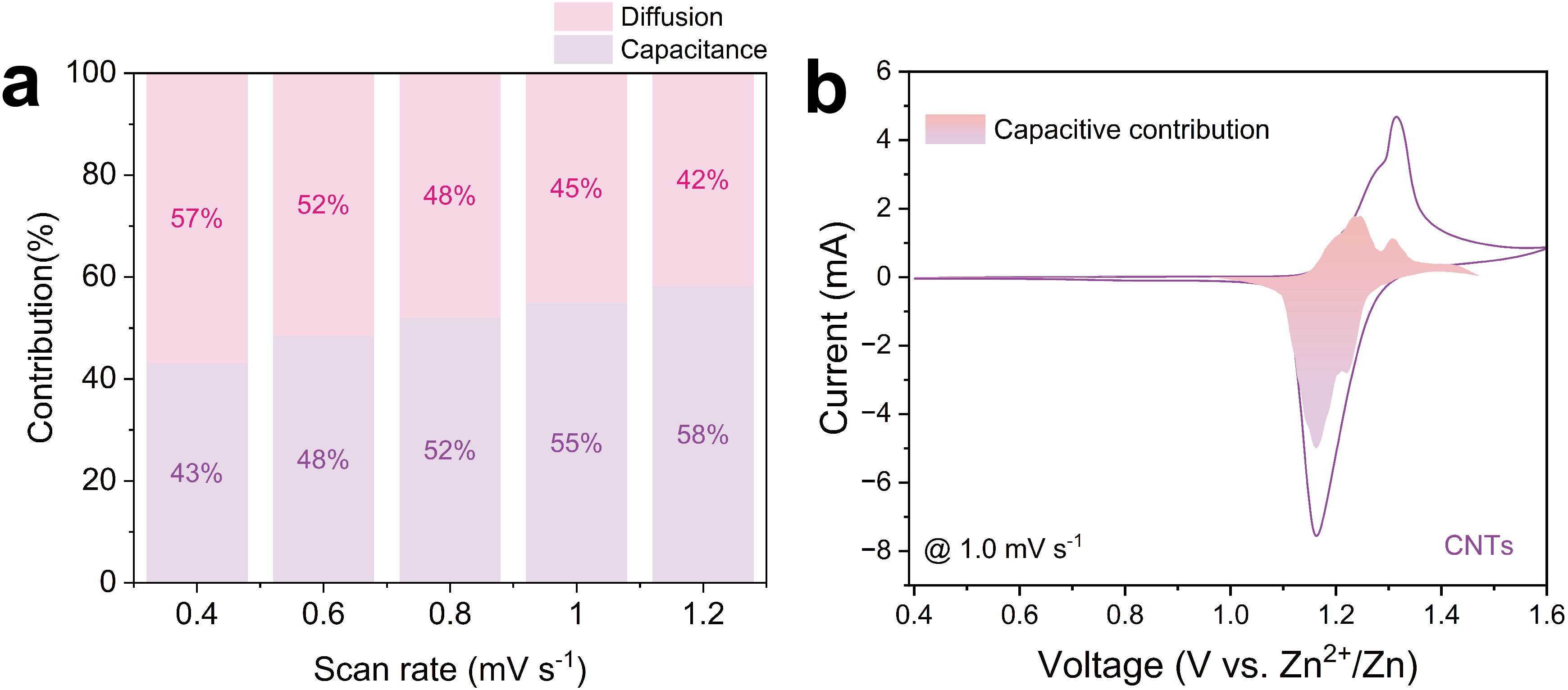


**Fig. S18** (**a**) Normalized capacitive contribution ratios of carboxyl-CNTs cathode at different scan rates. (**b**) Capacitive behaviors contributions of carboxyl-CNTs at 1.0 mV s^–1^


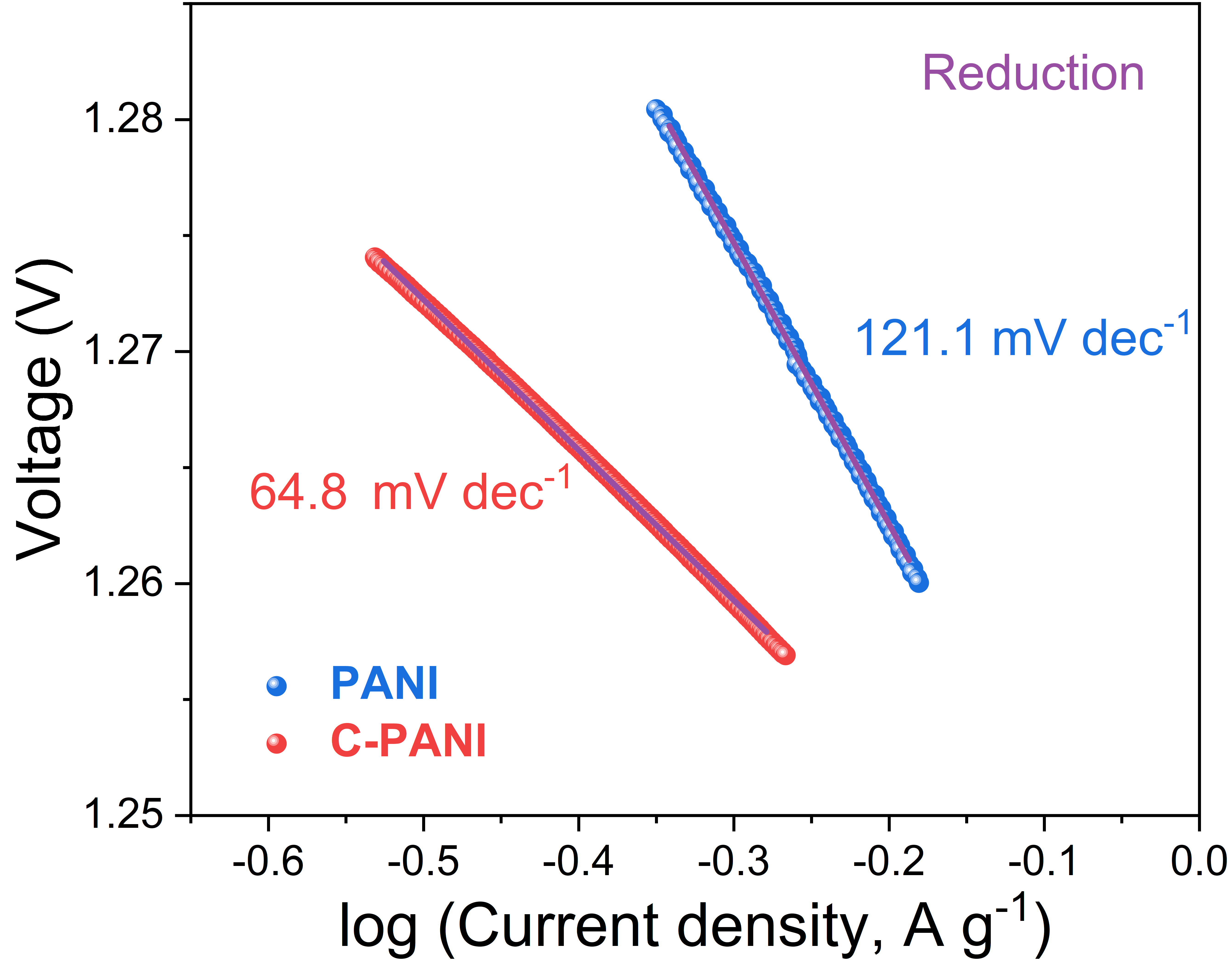


**Fig. S19** Tafel plots of C-PANI and PANI cathodes during the iodine reduction process, respectively


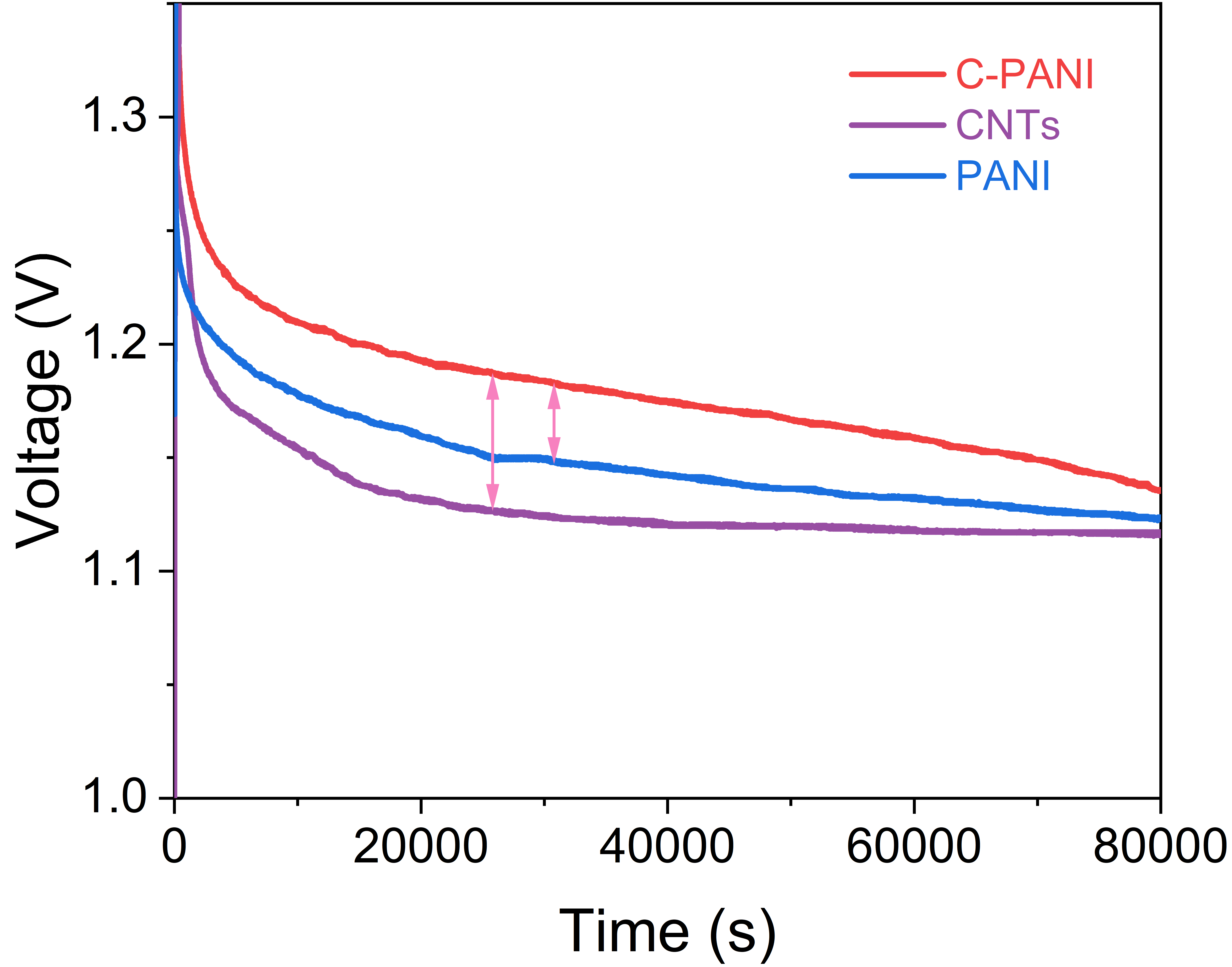


**Fig. S20** Self-discharge performances over 80000 s


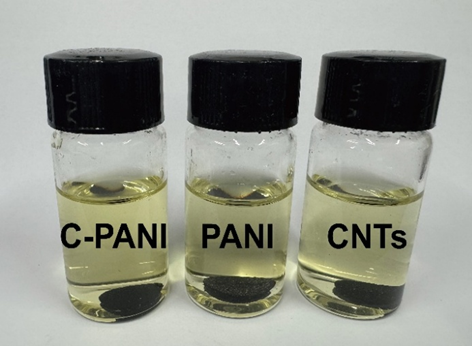


**Fig. S21** C-PANI, PANI and carboxyl-CNTs cathodes were immersed into 0.3 M NH_4_I solution at the initial state





**Fig. S22** *In-situ* EIS plots of carboxyl-CNTs cathode during the initial two cycles in 2 M Zn(OTF)_2_/0.3 M NH_4_I electrolyte


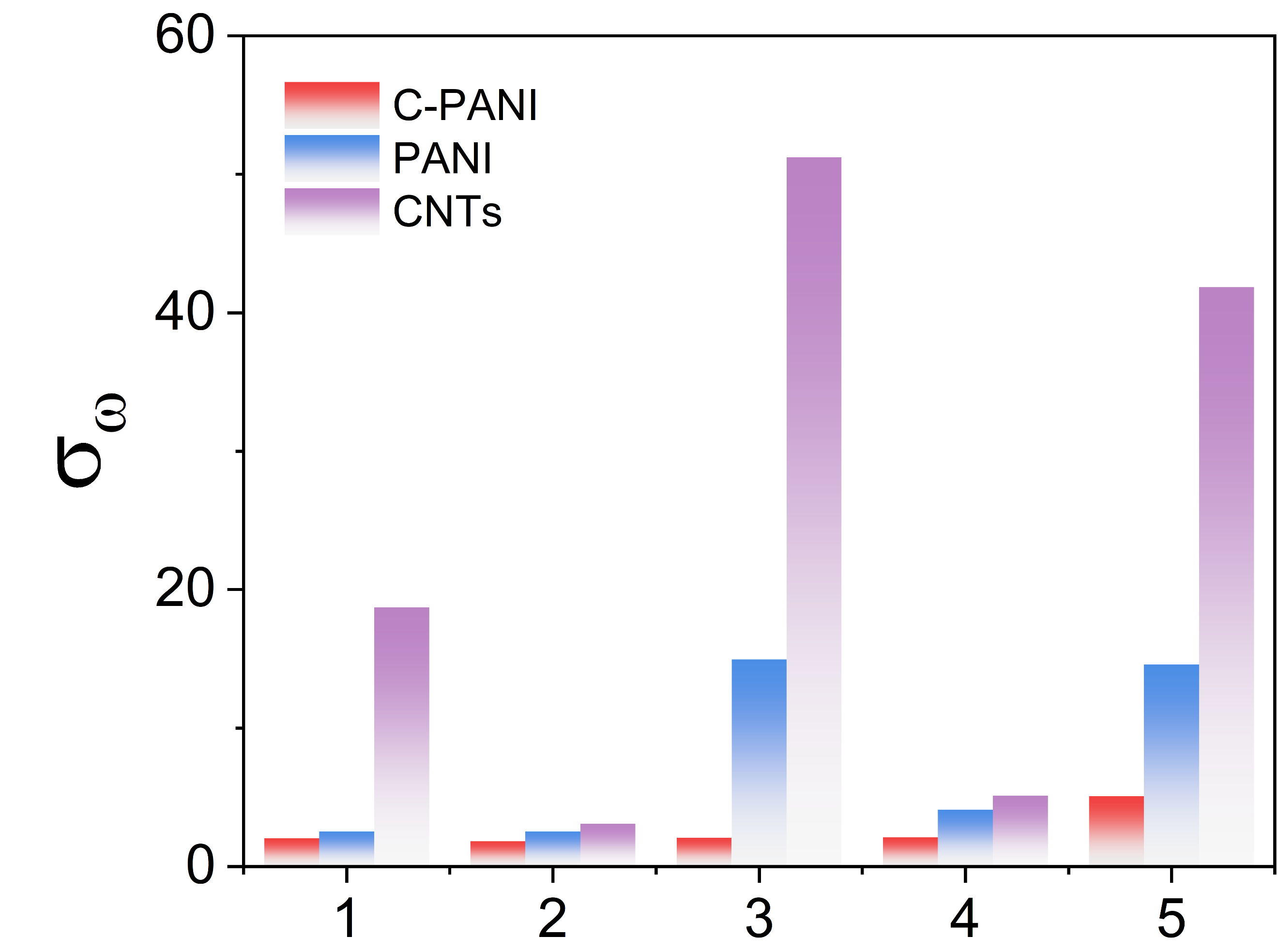


**Fig. S23** corresponding calculated impedance data of Warburg impedance. (States 1 through 5 represent the initial state, the first cycle fully-charged state, the first cycle fully-discharged state, the second cycle fully-charged state, and the second cycle fully-discharged state, respectively)

**
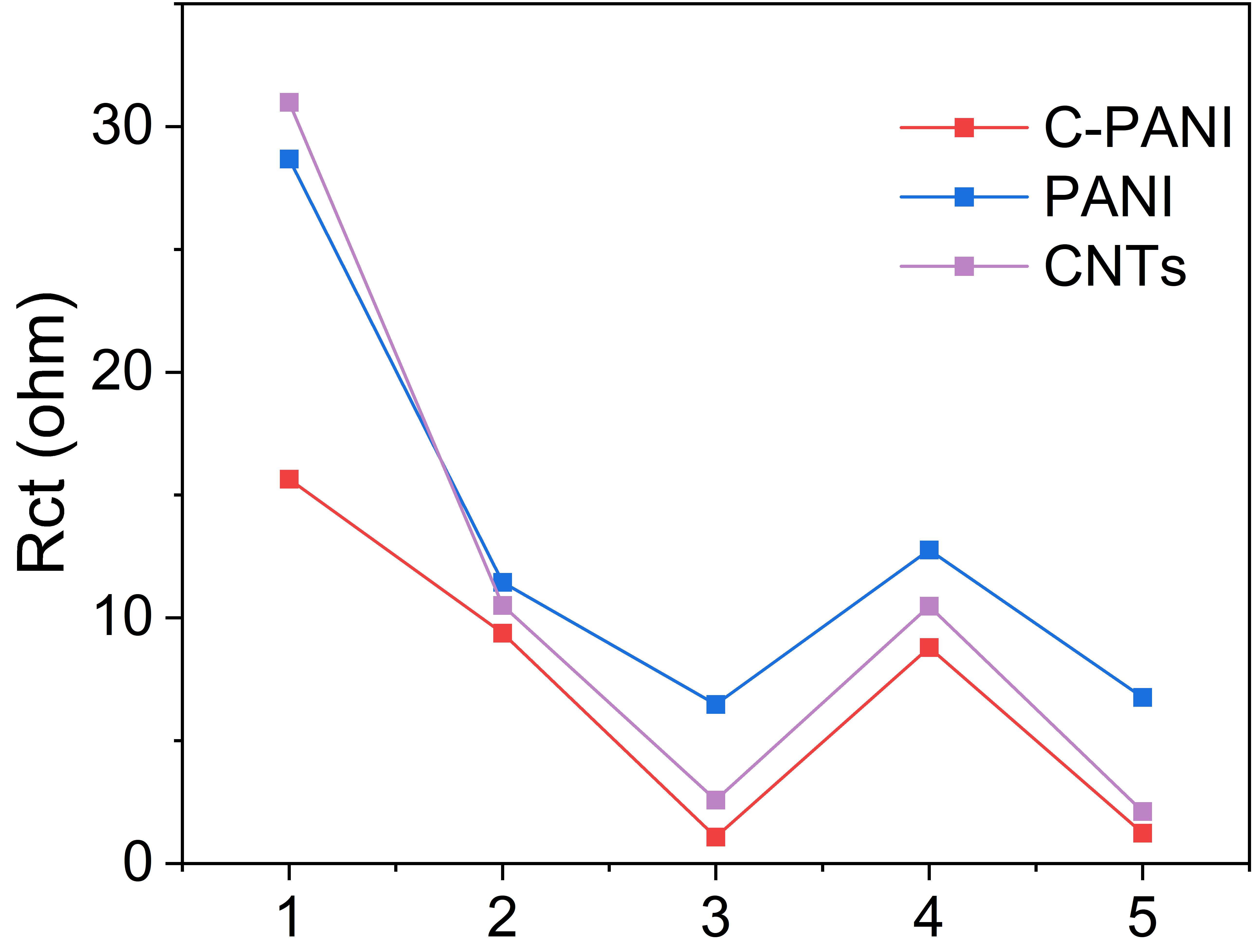
**

**Fig. S24** corresponding calculated impedance data of Rct. (States 1 through 5 represent the initial state, the first cycle fully-charged state, the first cycle fully-discharged state, the second cycle fully-charged state, and the second cycle fully-discharged state, respectively)


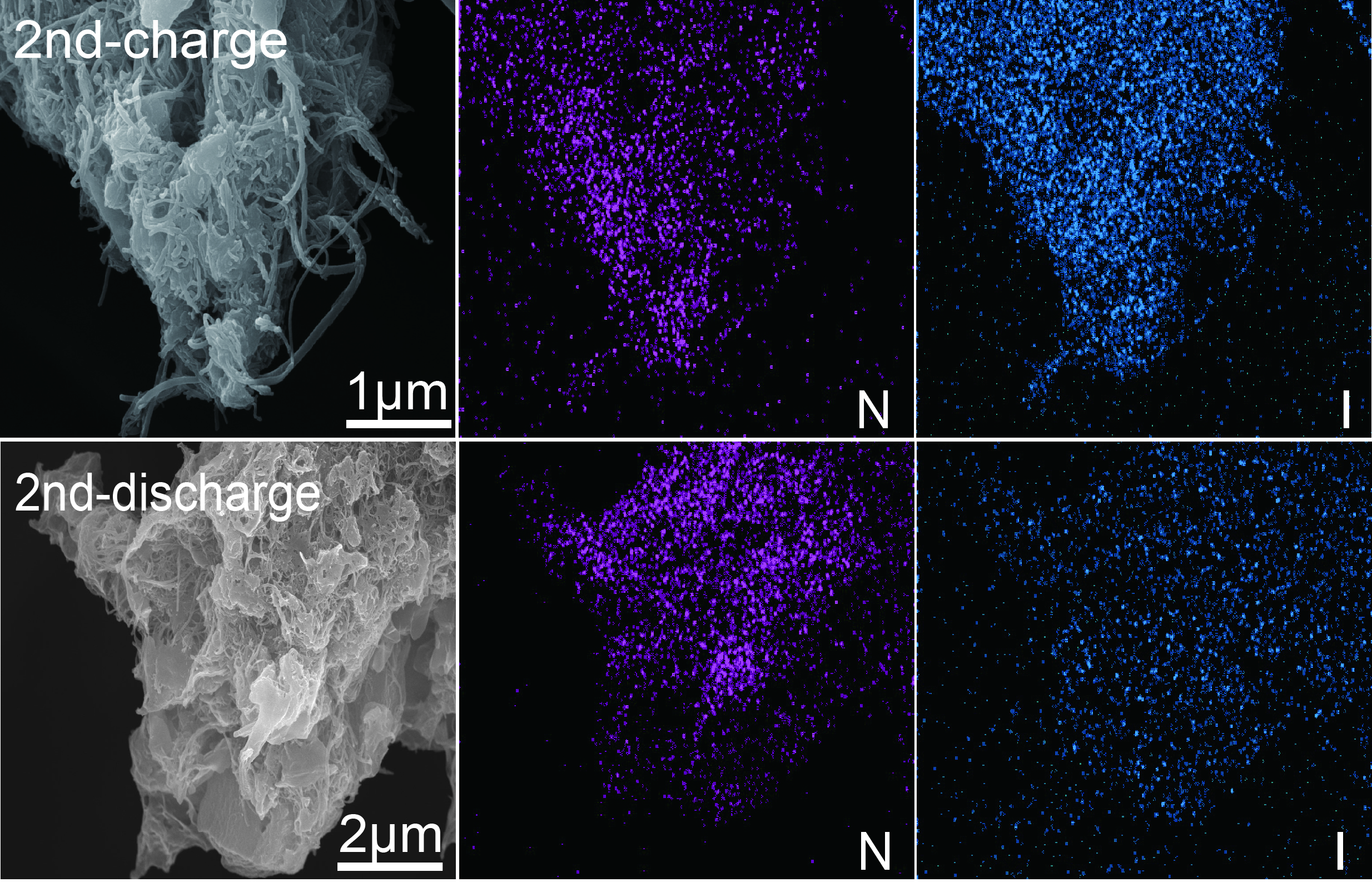


**Fig. S25** SEM images of C-PANI cathode in different charging and discharging states


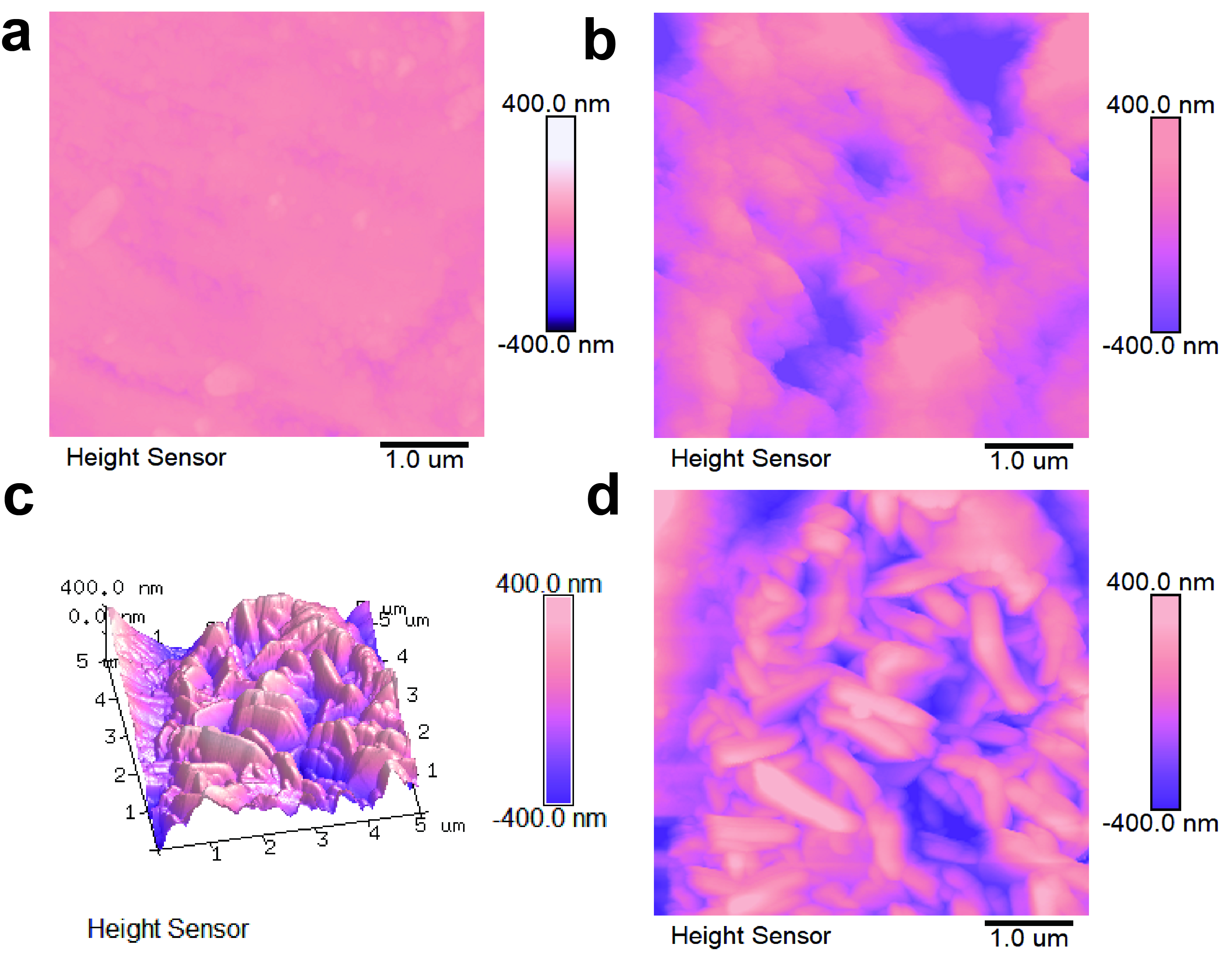


**Fig. S26** Two-dimensional AFM images of cycled Zn anode matched with C-PANI (**a**) and PANI (**b**) respectively. Three-dimensional (**c**) and Two-dimensional (**d**) AFM images of cycled Zn anode matched with carboxyl-CNTs


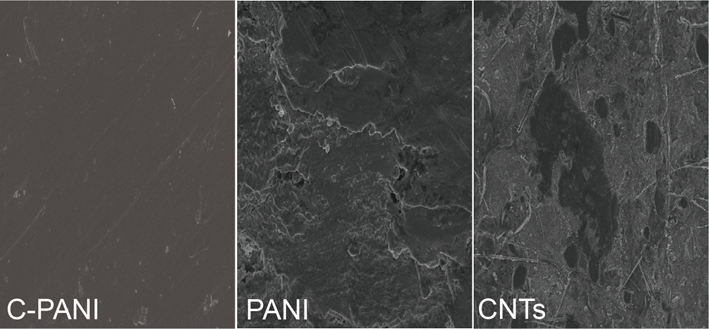


**Fig. S27** SEM images of Zn anodes matched with C-PANI, PANI, and CNTs cathodes, respectively

**
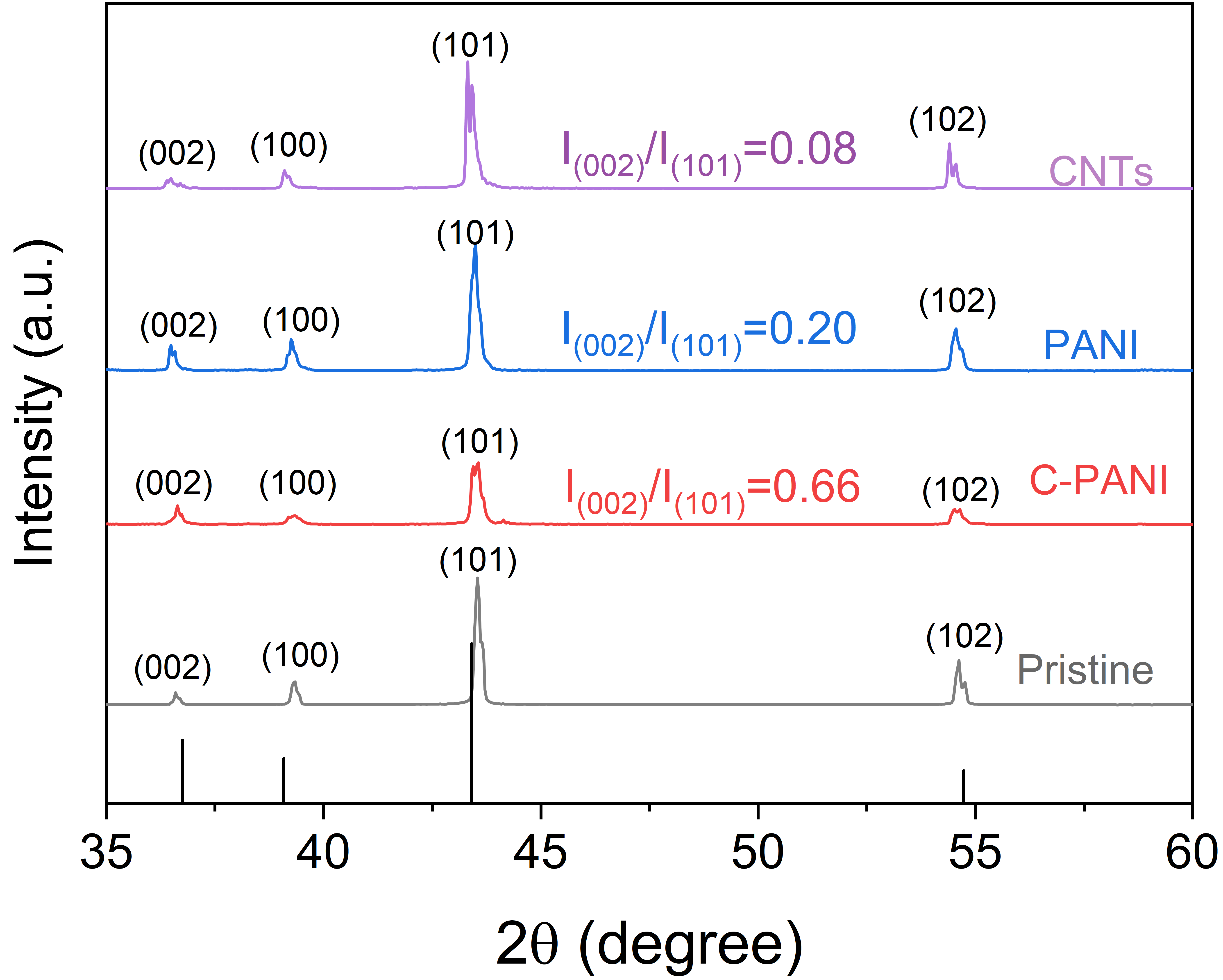
**

**Fig. S28** XRD patterns of 100^th^-cycled Zn anodes matched with C-PANI, PANI, and CNTs cathodes, respectively, at 2.0 A g^−1^


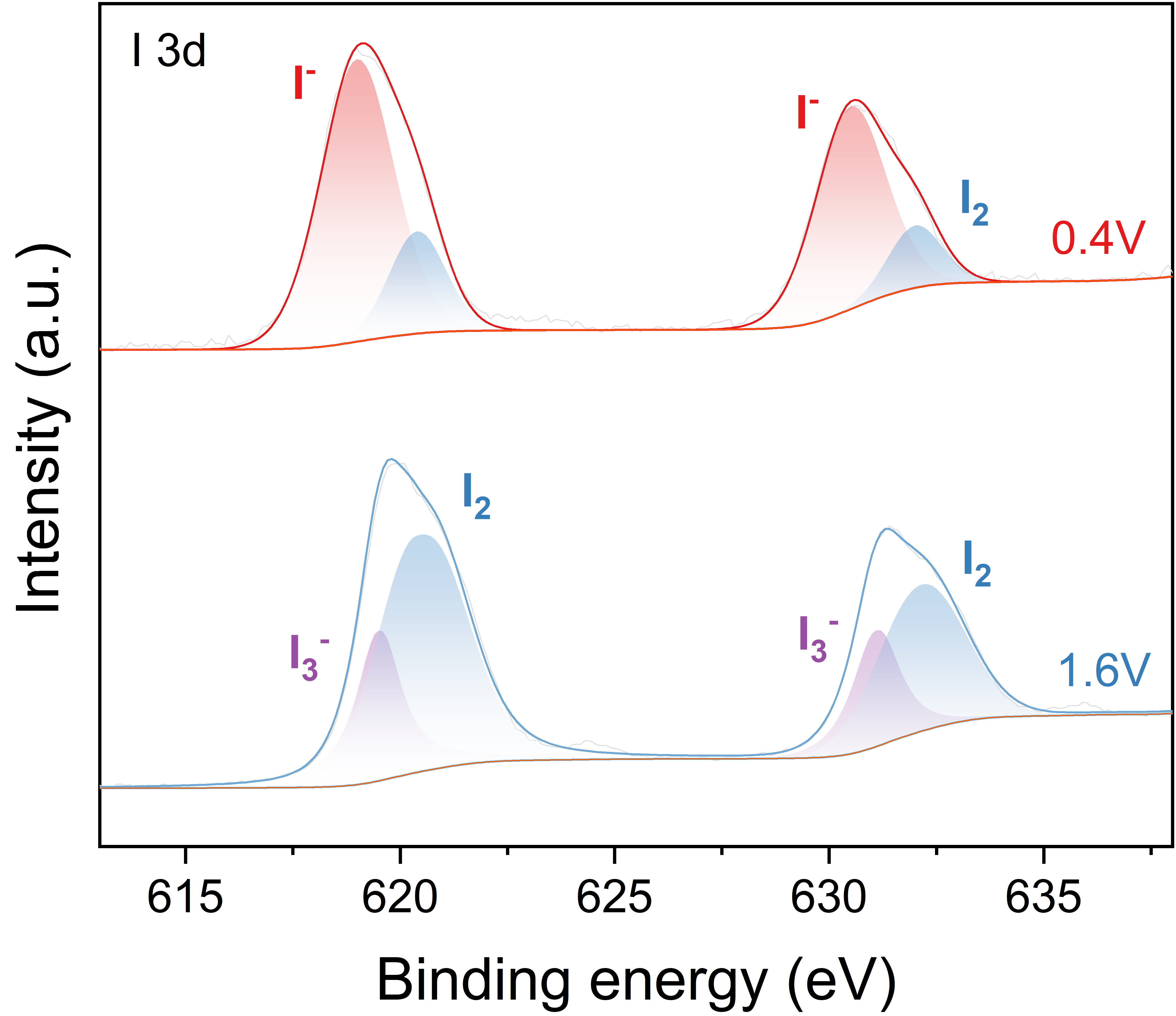


**Fig. S29** *Ex-situ* spectra for the PANI of high-resolution XPS I 3d at different working states


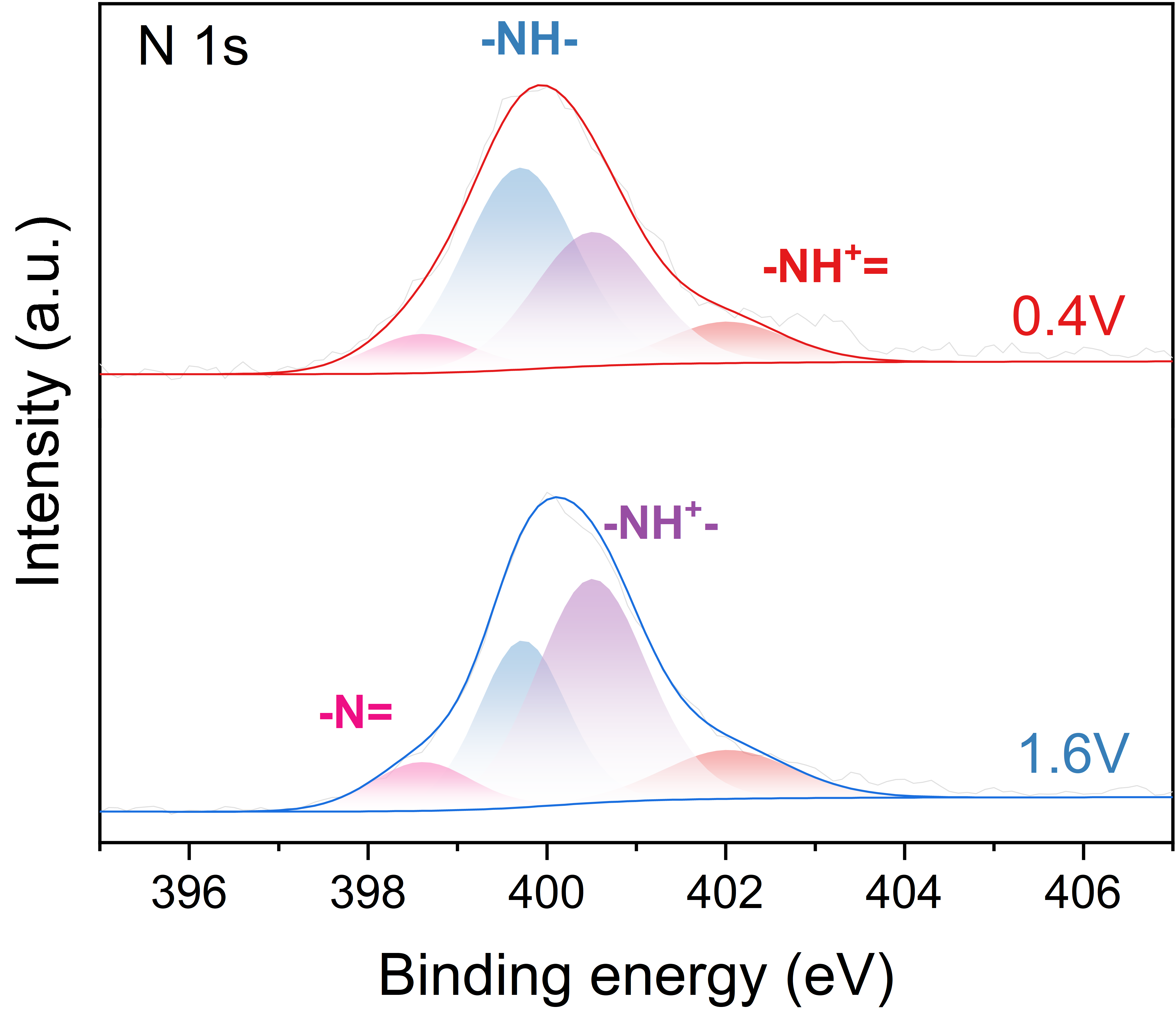


**Fig. S30** *Ex-situ* spectra for the PANI of high-resolution XPS N 1s at different working states


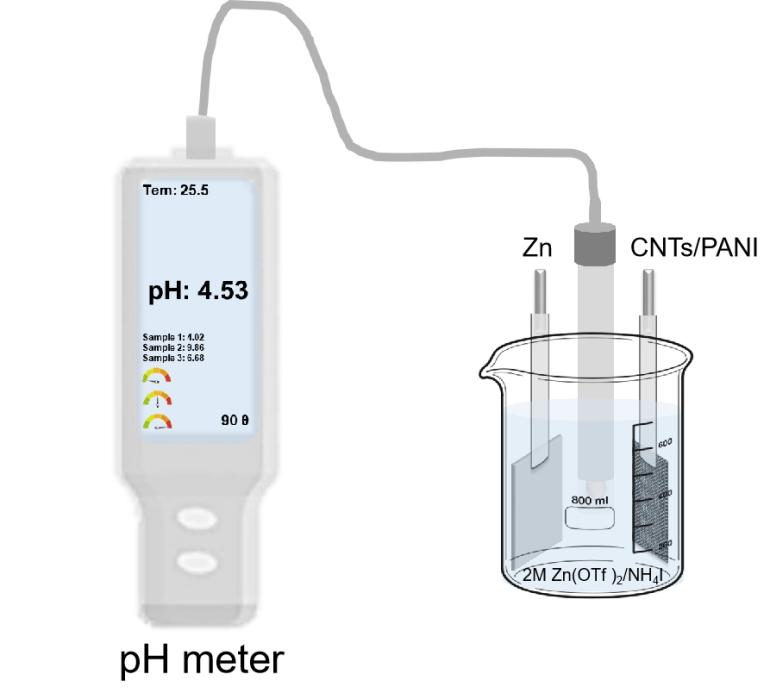


**Fig. S31** Diagram of in-situ pH test. Place the cathode (C-PANI) and the anode (zinc foil) into the electrolytic cell, add a certain amount of electrolyte (2 M Zn(OTF)_2_ + 0.3 M NH_4_I), phInsert the pH meter near the position of the cathode to monitor the changes in pH within the electrolyte under various charging and discharging states


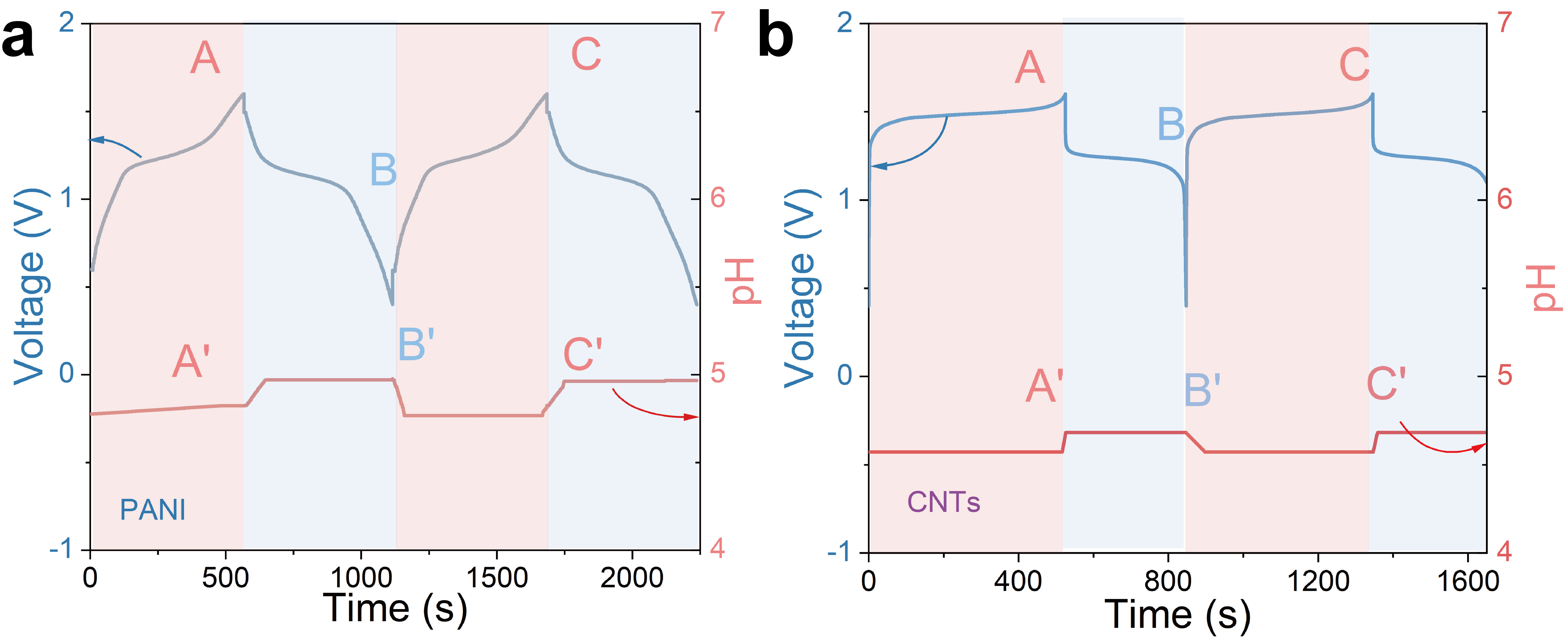


**Fig. S32** *In-situ* PH changes of aqueous Zn–I_2_ batteries at the (**a**) PANI and (**b**) CNTs side, respectively, in 2 M Zn(OTF)_2_ and 0.3 M NH_4_I electrolytes

In 2 M Zn(OTF)_2_ and 0.3 M NH_4_I electrolyte, the *in-situ* pH tests of electrolytic Zn-I_2_ batteries at the cathode sides was conducted using pure carboxyl-CNTs and PANI as catalytic cathodes, respectively. As a result, the pH values at this two cathodes side decreased during the charging process, attributing to the proton release from the cathode into the electrolytes (**Fig. S32**). During the following discharging process, the proton ions were inserted and combined with the cathode materials to cause the pH increase. Notably, catalytic PANI cathode exhibits a higher proton storage capacity than that of carboxyl-CNTs, which leads to relatively greater pH variation throughout the Zn-I_2_ battery working.

However, when C-PANI was employed as the cathode material, the carboxyl-CNTs function as a proton reservoir to supply protons to PANI, thereby establishing a dynamic pH equilibrium during battery cycling (**Fig. 4e**). This enables the realization of a “proton-iodine” synergistical regulation for the efficient iodine adsorption and direct I^−^/I^0^ conversion in aqueous Zn-I_2_ battery.


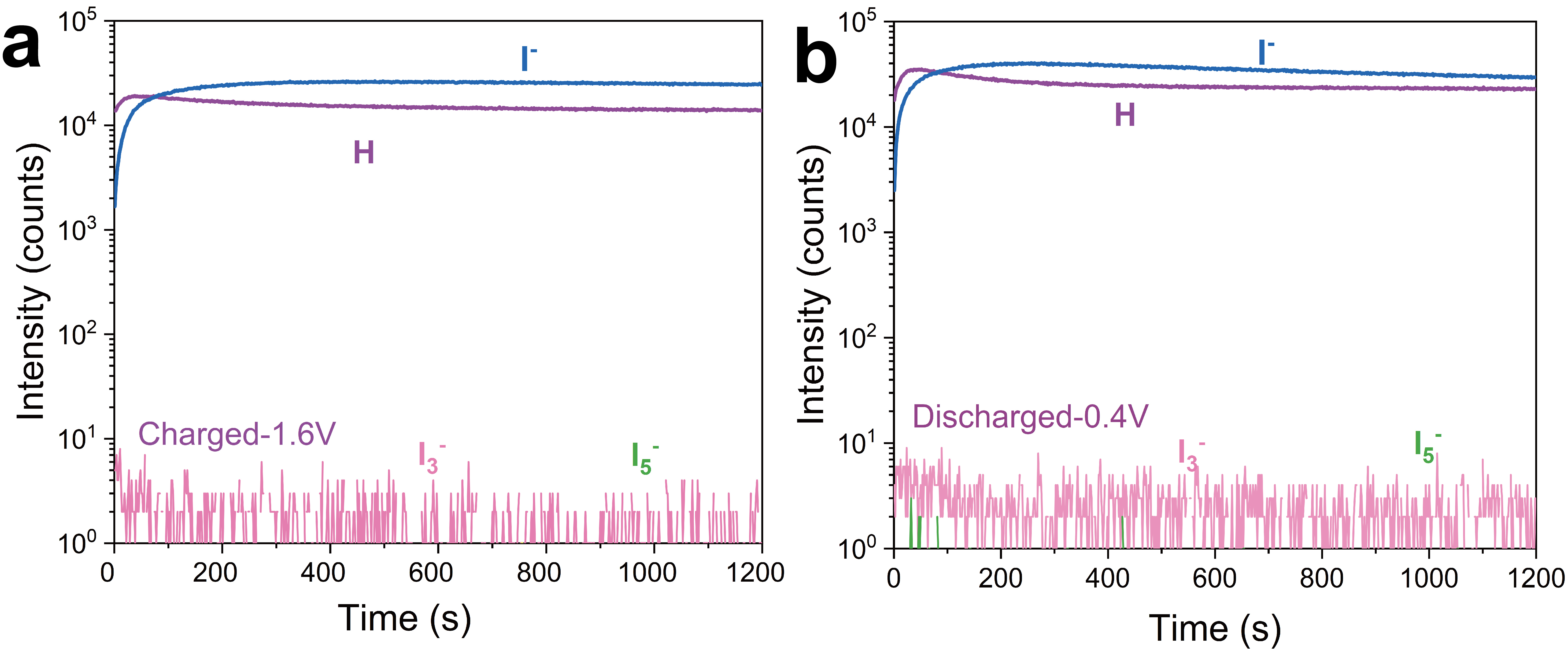


**Fig. S33** Depth profiling TOF-SlMS analyses of C-PANI cathode at fully discharged (**a**) and charged (**b**) states after 10 cycles





**Fig. S34** 3D distribution analysis from the TOF-SIMS depth-scan of the C-PANI electrode at the charged to 1.6 V state and discharged to 0.4 V state

**

**

**Fig. S35** Upside: Isosurface map of ESP for initial PANI (isovalue=0.05 au). Blue and red isosurfaces correspond to positive and negative parts of ESP, respectively. Downside: HOMOs and LUMOs of initial PANI


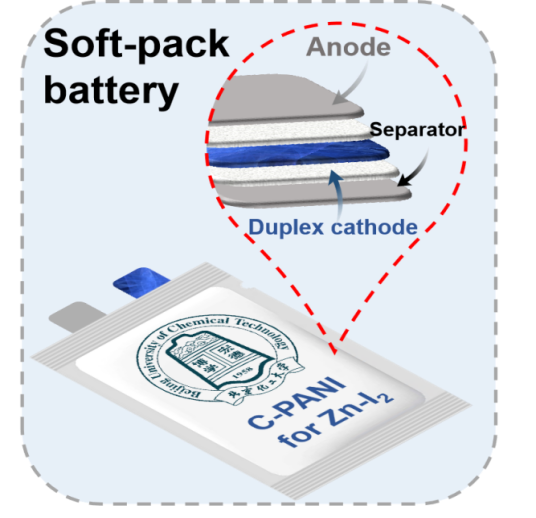


**Fig. S36** Schematic diagram for the assemble process of the flexible Zn-I_2_ battery

**
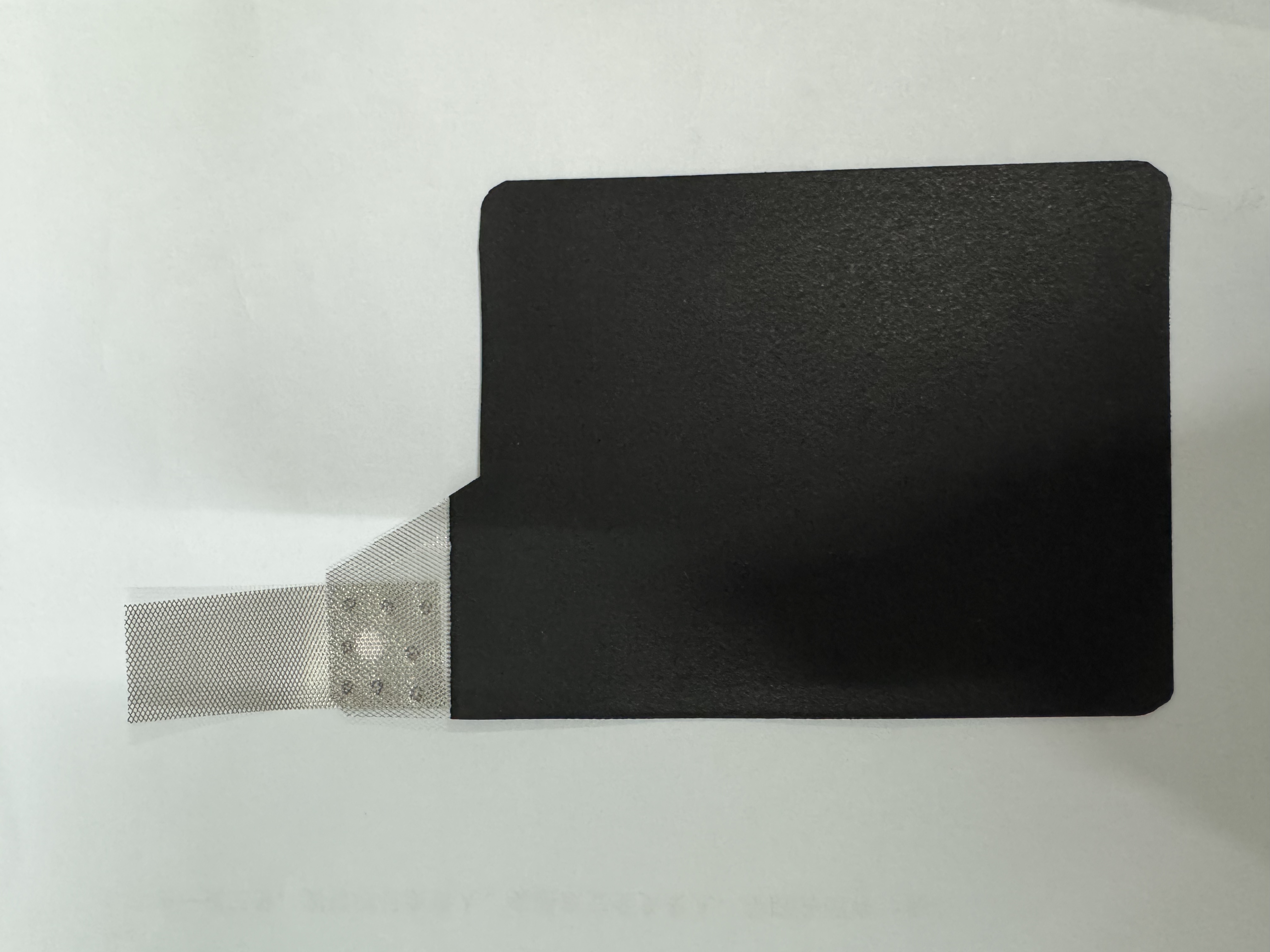
**

**Fig. S37** The high-mass C-PANI cathode (21.5 mg cm^–2^) based on a stainless-steel mesh current collector


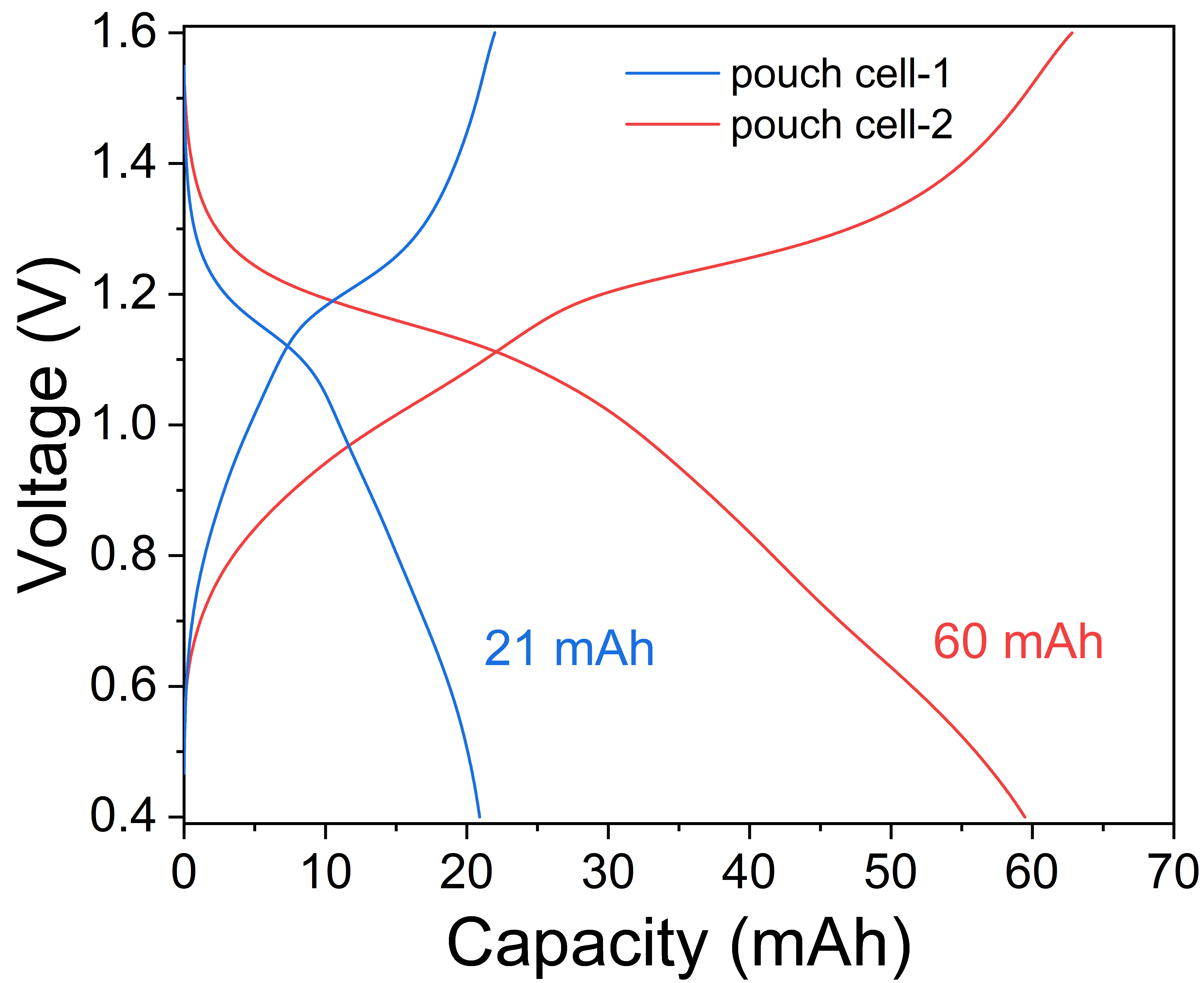


**Fig. S38** GCD curves of different sizes of pouch cell


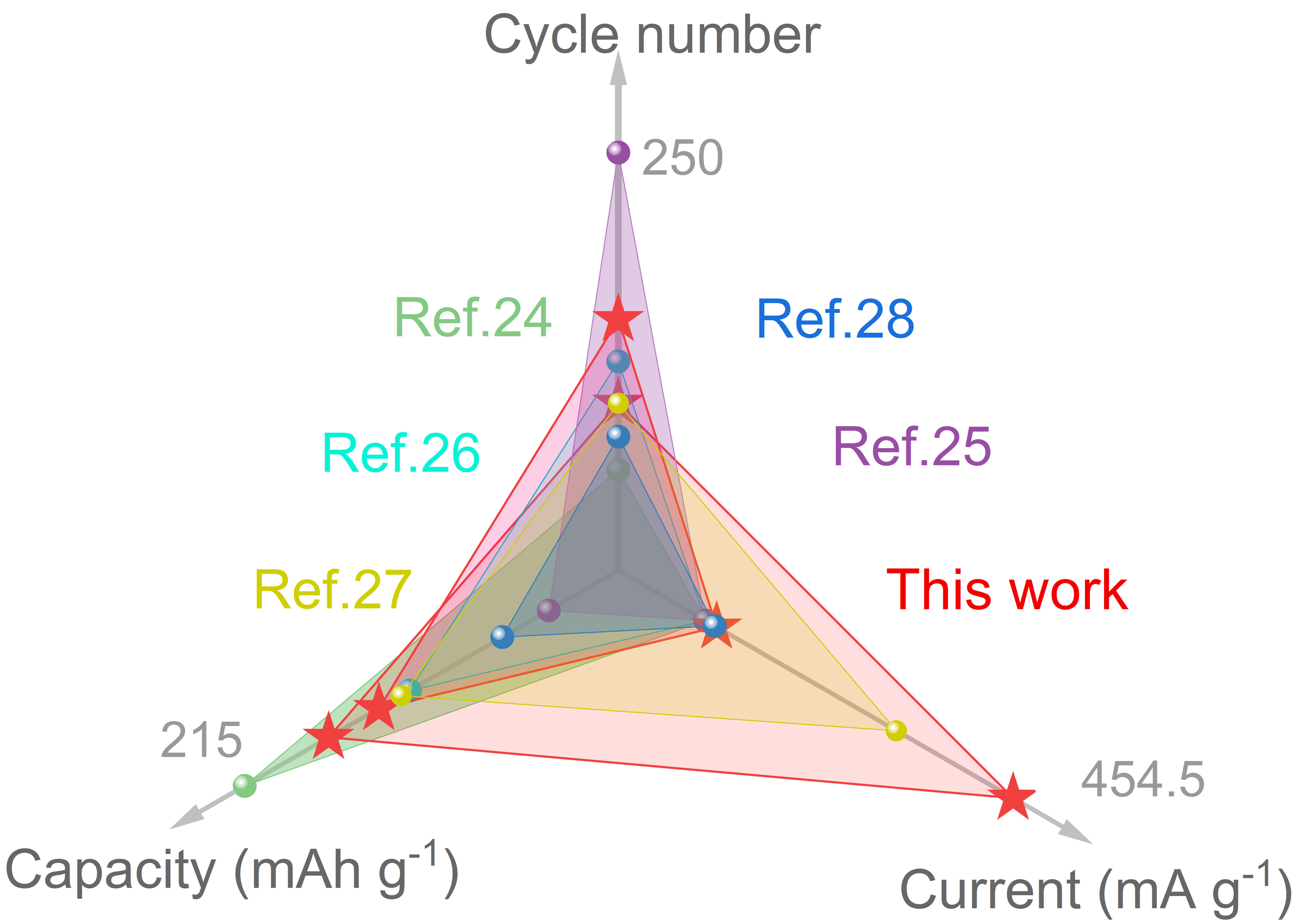


**Fig. S39** Comparison of the cycle number, current density, and capacity of this work with other iodine batteries reported in the literature


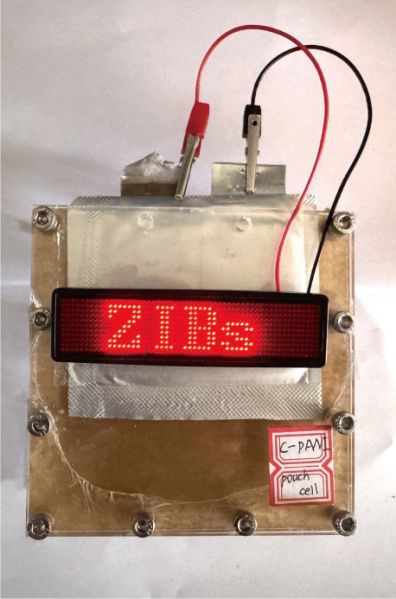


**Fig. S40** Device demonstrations of light-emitting diode (LED) board powered by the pouch cells

**Table S1** Battery performance comparisons of catalytic C-PANI and other cathodes.

| **Cathode** | **Electrolyte** | **Capacity** | **Cycle retention** | **Ref.** |
| --- | --- | --- | --- | --- |
| PANI-I_2_ | 2 M ZnSO_4_ | 230 mAh g^-1^ @ 0.3 A g^-1^ | 79% after 700 cycles at 1.5 A g^-1^ | [S11] |
| GC-PAN/I | 2 M ZnSO_4_ | 146.1 mAh g^-1^ @ 1 C,  133.8 mAh g^-1^ at 10 C | 97.24% after 2000 cycles at 20 C | [S12] |
| Zn-PymSH | 2 M ZnSO_4_+  0.5 M KI | 207 mAh g^-1^ at 5 A g^-1^ | ~100% after 5000 cycles at 5 A g^-1^ | [S13] |
| Zn-TCPP | 3 M ZnSO_4_+  0.5 M KI | 278 mAh g^-1^ at 5 A g^-1^ | ~100% after 5000 cycles at 5 A g^-1^ | [S14] |
| ppy@CF | 2 M ZnSO_4_ | 160.9 mAh g^−1^ at 0.5 C | ~ 100 % after 17000 cycles at 20C | [S15] |
| PANI-PEA | ZnSO_4_-PEA | 105 mAh g^−1^ at 2 A g^−1^ | 100 % after 800 cycles at 2 A g^-1^ | [S16] |
| PANI-GBL | 1 M Zn(CF_3_SO_3_)_2_ | 145 mAh g^-1^ at 0.5 A g^-1^ | 94 % after 200 cycles at 0.5 A g^-1^ | [S17] |
| PANI@CF | 1.5 M KI | 236 mAh g^-1^ at 0.5 A g^-1^ | 75.7 % after 10000 cycles at 2.0 A g^-1^ | [S18] |
| PANI | ZnI_2_/ZnCl_2_ | 2 mAh cm^-2^ @ 6 mA cm^-2^ | 99 % after 1000 cycles at 6 mA cm^-2^ | [S19] |
| PANI-S | 1 M ZnSO_4_ | 180 mAh g^-1^ at 0.1 A g^−1^ 121 mAh g^−1^ at 1.6A g^−1^ | 70% after2000 cycles at 0.2 A g^-1^ | [S20] |
| PANI | BMII−ZnSO_4_ | 895 μAh cm^-2^  at 2 mA cm^-2^ | 80% after 2800 cycles at 5mA cm^-2^ | [S21] |
| PANI-PAGE | 3 M Zn(ClO_4_)  -PAGE | 79.6 mAh g^-1^ at 15 A g^-1^ | 91.2% after 20000 cycles at 15 A g^-1^ | [S22] |
| S-Ti_3_C_2_T_x_  /PANI | 2 M ZnSO_4_ | 262 mAh g^-1^ at 0.5 A g^-1^  160 mAh g^-1^ at 15 A g^-1^ | 90% after 5000 cycles at 15 A g^-1^ | [S23] |
| **C-PANI** | **2 M Zn(CF_3_SO_3_)_2_ + 0.3 M NH_4_I** | **400 mAh g^-1^ at 2 A g^-1^ 260 mAh g^-1^ at 20 A g^-1^** | **95% after 5000 cycles at 3 A g^-1^**  **85.7% after 40,000 cycles at 20 A g^-1^** | **This work** |

**Table S2** Battery performance comparisons of reported Zn pouch cells.

| **Cathode** | **Current (mA g^-1^**) | | **Capacity (mAh g^-1^)** | **Cycle number** | **References** |
| --- | --- | --- | --- | --- | --- |
| MX-AB@I | | 100 | 120 | 125 | [S24] |
| NC-Co/ZnI_2_ | | 100 | 40 | 250 | [S25] |
| TEMPO | | 111.1 | 66.7 | 80 | [S26] |
| GC-PAN/I | | 320 | 125 | 100 | [S27] |
| Fe-N-C/I_2_ | | 100 | 215 | 60 | [S28] |
| C-PANI | | 113.2 | 137.74 | 150 | **This work** |
| C-PANI | | 454.5 | 166.67 | 100 | **This work** |

**Table S3** N/P ratios and energy density for different capacity of the electrolytic Zn//I_2_ pouch cells with C-PANI cathodes.

| **Data** | Pouch cell-1 | Pouch cell-2 |
| --- | --- | --- |
| **Capacity (mAh)** | 22 | 70 |
| **r_N/P_** | 33.02 | 25.06 |
| **M_1_ (g)** | 0.103 | 0.332 |
| **M_2_ (g)** | 0.980 | 2.445 |
| **Energy Density_1_ (Wh kg^−1^)** | 217.48 | 236.14 |
| **Energy Density_2_ (Wh kg^−1^)** | 24.44 | 28.63 |

The N/P ratio can be calculated using the following equation:

​$r_{N/P}= C_{\mathrm{Zn}}/C_{\mathrm{Cathode}}$

Pouch cell-1: $r_{N/P}=C_{\mathrm{Zn}}/C_{\mathrm{Cathode}}$ = (820 mAh g^−1^×0.886 g)/(22 mAh) = 33.02

Pouch cell-2: $r_{N/P}=C_{\mathrm{Zn}}/C_{\mathrm{Cathode}}$ = (820 mAh g^−1^×2.136 g)/(70 mAh) = 25.06

where C_Cathode_ represents the capacity of pouch cell at various mass loadings.

**Evaluation of energy density**

The gravimetric energy density of the assembled pouch-cell battery based on the mass iodine is critically assessed using the equation:

Pouch cell-1:$E_{g}=\frac{C(mAh)\times V(V)}{M_{1} (g)}$=$\frac{22 mAh\times1.12 V}{0.103 g}=217.48 \mathrm{Wh}\mathrm{kg}^{-1}$

$E_{g}=\frac{C(mAh)\times V(V)}{M_{2} (g)}$=$\frac{22 mAh\times1.12 V}{0.980 g}=24.44 \mathrm{Wh}\mathrm{kg}^{-1}$

Pouch cell-2: $E_{g}=\frac{C(mAh)\times V(V)}{M_{1} (g)}$=$\frac{70 mAh\times1.12 V}{0.332 g}=236.14 \mathrm{Wh}\mathrm{kg}^{-1}$

$E_{g}=\frac{C(mAh)\times V(V)}{M_{2} (g)}$=$\frac{70 mAh\times1.12 V}{2.445 g}=28.63 \mathrm{Wh}\mathrm{kg}^{-1}$

Where E_g_ denotes the full-cell gravimetric energy density Wh kg^−1^, V is the discharge average voltage for the Zn-I_2_ battery, recorded at 1.12 V, C reflects the capacity in mAh, and **M_1_ comprises the active material mass (g) of the pouch-cell, encompassing the cathode, M_2_ comprises the active material mass (g) of the pouch-cell, encompassing both anode and cathode.** The energy densities of 22 mAh and 70 mAh Zn-I_2_ pouch cells derived from active materials, particularly C-PANI, achieve the impressive values of 217.48 (24.44) and $236.14 ($28.63) Wh kg^−1^, respectively.

**Supplementary References**

1. M.J. Frisch, G.W. Trucks, H.B. Schlegel, et al., Gaussian 09 (Revision D.01). I. Gaussian, Wallingford, CT (2013).
2. J. Tirado-Rives, W.L. Jorgensen, Performance of B3LYP density functional methods for a large set of organic molecules. J. Chem. Theory Comput. **4**(2), 297–306 (2008). <https://doi.org/10.1021/ct700248k>
3. G. Kresse, J. Hafner, Ab initio molecular dynamics for liquid metals. Phys. Rev. B **47**(1), 558–561 (1993). <https://doi.org/10.1103/physrevb.47.558>
4. Kresse, G, &amp; Hafner, J, Ab initio molecular-dynamics simulation of the liquid-metal-amorphous-semiconductor transition in germanium. Phys. Rev. B Condens. Matter **49**(20), 14251–14269 (1994). <https://doi.org/10.1103/physrevb.49.14251>
5. J. Perdew, K. Burke, M. Ernzerhof, Generalized gradient approximation made simple. Phys. Rev. Lett. **77**(18), 3865–3868 (1996). <https://doi.org/10.1103/PhysRevLett.77.3865>
6. G. Kresse, D. Joubert, From ultrasoft pseudopotentials to the projector augmented-wave method. Phys. Rev. B **59**(3), 1758–1775 (1999). <https://doi.org/10.1103/physrevb.59.1758>
7. S. Grimme, J. Antony, S. Ehrlich, H. Krieg, A consistent and accurate *ab initio* parametrization of density functional dispersion correction (DFT-D) for the 94 elements H-Pu. J. Chem. Phys. **132**(15), 154104 (2010). <https://doi.org/10.1063/1.3382344>
8. J. Zhang, Y. Liu, X. Zhao, L. He, H. Liu et al., A novel NASICON-type Na_4_MnCr(PO4)_3_ demonstrating the energy density record of phosphate cathodes for sodium-ion batteries. Adv. Mater. **32**(11), 1906348 (2020). <https://doi.org/10.1002/adma.201906348>
9. J. Wang, H. Jia, Z. Liu, J. Yu, L. Cheng et al., Anchoring π-d conjugated metal–organic frameworks with dual-active centers on carbon nanotubes for advanced potassium-ion batteries. Adv. Mater. **36**(6), 2305605 (2024). <https://doi.org/10.1002/adma.202305605>
10. Y. Zou, T. Liu, Q. Du, Y. Li, H. Yi et al., A four-electron Zn-I2 aqueous battery enabled by reversible I–/ I2/I+ conversion. Nat. Commun. **12**, 170 (2021). <https://doi.org/10.1038/s41467-020-20331-9>
11. X. Zeng, X. Meng, W. Jiang, J. Liu, M. Ling et al., Anchoring polyiodide to conductive polymers as cathode for high-performance aqueous zinc–iodine batteries. ACS Sustainable Chem. Eng. **8**(38), 14280–14285 (2020). <https://doi.org/10.1021/acssuschemeng.0c05283>
12. L. Zhang, M. Zhang, H. Guo, Z. Tian, L. Ge et al., A universal polyiodide regulation using quaternization engineering toward high value-added and ultra-stable zinc-iodine batteries. Adv. Sci. **9**(13), 2105598 (2022). <https://doi.org/10.1002/advs.202105598>
13. Y. Tan, Z. Tao, Y. Zhu, Z. Chen, A. Wang et al., Anchoring I3–*via* charge-transfer interaction by a coordination supramolecular network cathode for a high-performance aqueous dual-ion battery. ACS Appl. Mater. Interfaces **14**(42), 47716–47724 (2022). <https://doi.org/10.1021/acsami.2c12962>
14. Y. Tan, Z. Chen, Z. Tao, A. Wang, S. Lai et al., A two-dimensional porphyrin coordination supramolecular network cathode for high-performance aqueous dual-ion battery. Angew. Chem. Int. Ed. **62**(12), e202217744 (2023). <https://doi.org/10.1002/anie.202217744>
15. D. Wang, N. Zhang, Y. Zhang, L. Chang, H. Tang et al., Electric field sponge effect of conducting polymer interphases boosts the kinetics and stability of zinc metal anodes. Adv. Energy Mater. **15**(11), 2404090 (2025). <https://doi.org/10.1002/aenm.202404090>
16. M. Wang, Y. Cheng, H. Zhao, J. Gao, J. Li et al., A multifunctional organic electrolyte additive for aqueous zinc ion batteries based on polyaniline cathode. Small **19**(29), 2302105 (2023). <https://doi.org/10.1002/smll.202302105>
17. S.-J. Zhang, J. Hao, Y. Zhu, H. Li, Z. Lin et al., pH-triggered molecular switch toward texture-regulated Zn anode. Angew. Chem. Int. Ed. **62**(17), e202301570 (2023). <https://doi.org/10.1002/anie.202301570>
18. X. Miao, Q. Chen, Y. Liu, X. Zhang, Y. Chen et al., Performance comparison of electro-polymerized polypyrrole and polyaniline as cathodes for iodine redox reaction in zinc-iodine batteries. Electrochim. Acta **415**, 140206 (2022). <https://doi.org/10.1016/j.electacta.2022.140206>
19. W. Wu, C. Li, Z. Wang, H.-Y. Shi, Y. Song et al., Electrode and electrolyte regulation to promote coulombic efficiency and cycling stability of aqueous zinc-iodine batteries. Chem. Eng. J. **428**, 131283 (2022). <https://doi.org/10.1016/j.cej.2021.131283>
20. H.-Y. Shi, Y.-J. Ye, K. Liu, Y. Song, X. Sun, A long-cycle-life self-doped polyaniline cathode for rechargeable aqueous zinc batteries. Angew. Chem. Int. Ed. **57**(50), 16359–16363 (2018). <https://doi.org/10.1002/anie.201808886>
21. R. Yang, W. Yao, L. Zhou, F. Zhang, Y. Zheng et al., Secondary amines functionalized organocatalytic iodine redox for high-performance aqueous dual-ion batteries. Adv. Mater. **36**(23), 2314247 (2024). <https://doi.org/10.1002/adma.202314247>
22. D. Feng, Y. Jiao, P. Wu, Proton-reservoir hydrogel electrolyte for long-term cycling Zn/PANI batteries in wide temperature range. Angew. Chem. Int. Ed. **62**(1), e202215060 (2023). <https://doi.org/10.1002/anie.202215060>
23. Y. Liu, Z. Dai, W. Zhang, Y. Jiang, J. Peng et al., Sulfonic-group-grafted Ti_3_C_2_T*_x_* MXene: a silver bullet to settle the instability of polyaniline toward high-performance Zn-ion batteries. ACS Nano **15**(5), 9065–9075 (2021). <https://doi.org/10.1021/acsnano.1c02215>
24. X. Guo, H. Xu, Y. Tang, Z. Yang, F. Dou et al., Confining iodine into metal-organic framework derived metal-nitrogen-carbon for long-life aqueous zinc-iodine batteries. Adv. Mater. **36**(38), 2408317 (2024). <https://doi.org/10.1002/adma.202408317>
25. D. Shen, A.M. Rao, J. Zhou, B. Lu, High-potential cathodes with nitrogen active centres for quasi-solid proton-ion batteries. Angew. Chem. Int. Ed. **61**(22), e202201972 (2022). <https://doi.org/10.1002/anie.202201972>
26. N.S.W. Gamage, Y. Shi, C.J. Mudugamuwa, J. Santos-Peña, D.A. Lewis et al., Converting a low-cost industrial polymer into organic cathodes for high mass-loading aqueous zinc-ion batteries. Energy Storage Mater. **72**, 103731 (2024). <https://doi.org/10.1016/j.ensm.2024.103731>
27. L. Zhang, M. Zhang, H. Guo, Z. Tian, L. Ge et al., A universal polyiodide regulation using quaternization engineering toward high value-added and ultra-stable zinc-iodine batteries. Adv. Sci. **9**(13), 2105598 (2022). <https://doi.org/10.1002/advs.202105598>
28. Y. Wang, X. Jin, J. Xiong, Q. Zhu, Q. Li et al., Ultrastable electrolytic Zn–I_2_ batteries based on nanocarbon wrapped by highly efficient single-atom Fe-NC iodine catalysts. Adv. Mater. **36**(30), 2404093 (2024). <https://doi.org/10.1002/adma.202404093>
